# Increased Risk of Hospital Admission for Asthma in Children From Short-Term Exposure to Air Pollution: Case-Crossover Evidence From Northern China

**DOI:** 10.3389/fpubh.2021.798746

**Published:** 2021-12-17

**Authors:** Yakun Zhao, Dehui Kong, Jia Fu, Yongqiao Zhang, Yuxiong Chen, Yanbo Liu, Zhen'ge Chang, Yijie Liu, Xiaole Liu, Kaifeng Xu, Chengyu Jiang, Zhongjie Fan

**Affiliations:** ^1^Department of Medicine, Peking Union Medical College Hospital, Peking Union Medical College, Chinese Academy of Medical Sciences, Beijing, China; ^2^National Key Laboratory of Medical Molecular Biology, Department of Biochemistry, Institute of Basic Medical Sciences, Peking Union Medical Colleges, Chinese Academy of Medical Sciences, Beijing, China

**Keywords:** air pollution, nitrogen dioxide (NO_2_), sulfur dioxide (SO_2_), childhood asthma, hospital admission, case-crossover

## Abstract

**Background:** Previous studies suggested that exposure to air pollution could increase risk of asthma attacks in children. The aim of this study is to investigate the short-term effects of exposure to ambient air pollution on asthma hospital admissions in children in Beijing, a city with serious air pollution and high-quality medical care at the same time.

**Methods:** We collected hospital admission data of asthma patients aged ≤ 18 years old from 56 hospitals from 2013 to 2016 in Beijing, China. Time-stratified case-crossover design and conditional Poisson regression were applied to explore the association between risk of asthma admission in children and the daily concentration of six air pollutants [particulate matter ≤ 2.5 μm (PM_2.5_), particulate matter ≤ 10 μm (PM_10_), sulfur dioxide (SO_2_), nitrogen dioxide (NO_2_), carbon monoxide (CO), and ozone (O_3_)], adjusting for meteorological factors and other pollutants. Additionally, stratified analyses were performed by age, gender, and season.

**Results:** In the single-pollutant models, higher levels of PM_2.5_, SO_2_, and NO_2_ were significantly associated with increased risk of hospital admission for asthma in children. The strongest effect was observed in NO_2_ at lag06 (*RR* = 1.25, 95%CI: 1.06-1.48), followed by SO_2_ at lag05 (*RR* = 1.17, 95%CI: 1.05–1.31). The robustness of effects of SO_2_ and NO_2_ were shown in two-pollutant models. Stratified analyses further indicated that pre-school children (aged ≤ 6 years) were more susceptible to SO_2_. The effects of SO_2_ were stronger in the cold season, while the effects of NO_2_ were stronger in the warm season. No significant sex-specific differences were observed.

**Conclusions:** These results suggested that high levels of air pollution had an adverse effect on childhood asthma, even in a region with high-quality healthcare. Therefore, it will be significant to decrease hospital admissions for asthma in children by controlling air pollution emission and avoiding exposure to air pollution.

## Introduction

Asthma is a common chronic respiratory disease without curable treatment. It has a substantial burden of disease, and ~273,000,000 people are afflicted worldwide (1). As the Global Burden of Disease Study reported, there were 495,100 deaths due to asthma at the global level and 22,800,000 disability-adjusted life years (DALYs) attributable to asthma in 2017 (2, 3). Compared with adults, children are more prone to suffering from asthma. According to the Global Burden of Disease Study, asthma approximately accounted for 2.29% of the global total DALYs among children aged 5–14 years in 2019 (4), implying that efficiently reducing childhood asthma hospital visits will bring about great public health benefits.

As a heterogeneous disease caused by genetic and environmental factors, asthma can be controlled by medicines and avoiding exposure to risk factors. In children, several environmental risk factors have been identified to be correlated with asthma exacerbation, such as viral infection, allergen exposure, cigarette smoking, and outdoor air pollution (5, 6). Children have relatively immature lungs and immune systems and inhale a larger volume of air per body weight (7), so they are more susceptible to the adverse respiratory effects of air pollution than adults.

Over the past few years, numerous researches showed that air pollution was associated with a significantly increased risk of outpatient and inpatient visits for asthma in children (8, 9). Even though the air quality has been improved in the past decades, air pollution remains one of the most serious public environmental problems worldwide. The effect of air pollution exposure on childhood asthma still requires constant attention.

As the capital of China, Beijing is one of the most economically developed cities with high-quality healthcare in China, and asthma was better controlled and had a lower hospitalization rate than other cities in China (10, 11). At the same time, Beijing is located in the northern region of China (39°56N, 116°20E), with a serious condition of air pollution. To reveal how air pollution affects childhood asthma attack on the condition of poor air quality and high-quality medical care, it will be worthwhile to explore the impact of air pollution on hospital admissions for asthma in children in Beijing.

This study aimed to investigate the short-term effects of ambient particulate matter ≤ 2.5 μm (PM_2.5_), particulate matter ≤ 10 μm (PM_10_), sulfur dioxide (SO_2_), nitrogen dioxide (NO_2_), carbon monoxide (CO), and ozone (O_3_) on hospital admissions for childhood asthma in Beijing, a city with poor air quality and high-quality medical care at the same time. Additionally, gender-, aged-, and season-stratified analyses were performed to explore susceptible populations and the potential effect of modification.

## Materials and Methods

### Study Population and Data Collection

This study enrolled all Beijing residents aged ≤ 18 years old who were admitted to a hospital in Beijing from 1st January 2013 to 31st December 2016 with a primary diagnosis of asthma (ICD-10, J45-46). The hospital admissions data were collected by the Beijing Municipal Health Commission Information Center (http://www.phic.org.cn/) from all public and private hospitals in Beijing. Each record includes hospital name, patient's gender, age, birthplace, current address, registration address, hospital admission date, the main discharge diagnoses, etc. All population data were collected anonymously in this study.

Air pollution data were obtained from China National Environmental Monitoring Centre. Continuous hourly concentrations of PM_2.5_, PM_10_, SO_2_, NO_2_, CO, and O_3_ were collected by 12 national air quality monitoring stations, which were widely distributed in Beijing and provided high-quality air monitoring data. Daily 24-h average concentrations were calculated for each pollutant. To control the effects of meteorological conditions, daily mean temperature (Tmean), relative humidity (RH), wind speed (WS), and air pressure (AP) were obtained from Beijing Meteorological Service (http://bj.cma.gov.cn/).

Many different respiratory viruses are associated with asthma exacerbations, and influenza viruses are frequently associated with those requiring hospitalization (12, 13). We adjusted influenza epidemic in this study, and influenza epidemic was defined as when the positive rate of influenza isolation in any given week exceeded 20% of the maximum weekly positive rate of influenza isolation in the whole surveillance season (from the 27th week of the previous year to the 26th week of the following year) in northern China (14). Data on influenza surveillance was accessed from the Chinese National Influenza Center (CNIC) (http://www.chinaivdc.cn/cnic/), which reports weekly influenza isolation positive rates in the northern and southern in China separately.

### Study Design

Case-crossover design has unique advantages in controlling time-invariant individual confounders such as individual metabolic and behavioral factors, as the cases themselves serve as their own controls (15). It has been widely used for investigating acute effects of air pollution on various health outcomes (16, 17).

A time-stratified case-crossover design was applied to assess the effect of short-term exposure to ambient air pollution on daily childhood asthma hospital admissions as a result of moderate and severe asthma attacks. In this study, the case day was defined as the date of asthma hospital admission. The control days were selected from the same day of the week within the same calendar year and month to control for potential confounding effects of day of the week, long-term trend and seasonality. This strategy resulted in 3 or 4 control days for each case. For example, if the case day (7th January 2013) was Monday, the remaining Mondays in the same month (14th, 21st, and 28th January 2013) would be matched as control days.

### Statistical Analysis

The correlations among the air pollutants and meteorological factors during the study period were analyzed using Spearman's correlation coefficient.

Conditional Poisson regression model, which has the advantage of readily allowing for overdispersion and autocorrelation (18), was used to evaluate the effect of exposure to air pollution on childhood asthma. In order to select the best-fit model, all meteorological factors, influenza epidemic, and public holiday were adjusted one by one in the following basic model:


$$\begin{array}{l} \text{Log}\left[\text{E}\left({\text{Y}}_{\text{t}}\right)\right]=\text{β}{\text{Z}}_{\text{t}}+\text{n}{\text{s}}_{\text{i}}\left({\text{X}}_{\text{i}},3\right)+\ldots .+\text{factor}\left({\text{X}}_{\text{j}}\right)+\text{intercept} \end{array}$$

where, t is the day of observation; Y_t_ refers to the number of hospital admissions for childhood asthma on day t; β refers to the vector of coefficients for Z_t_; Z_t_ refers to the daily average level of the air pollutant on day t; ns_i_(X_i_,3) indicates natural cubic spline with a fixed degree of freedom (df) 3 to control for non-linearity of meteorological factors X_i_; factor(X_j_) indicates a classification variable to adjust for the effect of influenza epidemic and public holiday; intercept is the constant term. We selected the final model based on the minimum Akaike information criterion for quasi-poisson (Q-AIC) value (19).

Based on the minimum Q-AIC value, the final single-pollutant model adjusted for daily mean temperature, influenza epidemic, and public holiday was shown as follows:


$$\begin{array}{l} \text{Log}\left[\text{E}\left({\text{Y}}_{\text{t}}\right)\right]=\text{β}{\text{Z}}_{\text{t}}+\text{ns}\left(\text{Tmean},\text{df}=3\right)+\text{factor}\left(\text{influenza}\right)+\text{factor}\left(\text{holiday}\right)+\text{intercept} \end{array}$$

where, t is the day of observation; Y_t_ refers to the number of childhood asthma admissions on day t; β refers to the vector of coefficients for Z_t_; Z_t_ refers to the daily average level of air pollutant on day t; ns() indicates natural cubic spline with 3 df to control for non-linearity of daily mean temperature; Tmean is daily mean temperature on the same day in the original model; factor(influenza) indicates a classification variable to adjust for the effect of influenza epidemic; factor(holiday) indicates a classification variable to adjust for the effect of public holiday; intercept is the constant term.

Previous studies have shown that air pollution has lag effects on asthma (20, 21). In this study, the single-day lags from the current day up to the previous 1-6 days (lag0 to lag6) and multi-day moving average lags of current and previous 1–6 days (lag01 to lag06) were applied to evaluate the lag effects of air pollutants. In this study, the strongest single lag day of a specific pollutant was the day with the strongest effect of this pollutant in single-day lag models. In other words, we obtained the strongest single lag day of each pollutant according to the maximum values of RRs when the RRs were >1. If the RRs of a pollutant were <1 and statistically significant, this pollutant's strongest single lag day was obtained according to the minimum values of RRs.

We performed stratified analysis by: (1) gender: male vs. female; (2) age: preschool children vs. school-aged children (age under 6 years as preschoolers, 7–18 years as school-aged children in most cities of China including Beijing); (3) season: cold season (from November to March of next year, during which the daily mean temperature was below 0°C at most time and the urban city burned coal for central heating) vs. warm season (from April to October). The lag effects (from lag0 to lag6 and lag01 to lag06) were also evaluated in stratified analyses. The statistical differences between effect estimates for stratified analyses were examined by calculating 95% confidence interval as shown below:


$$({\hat{Q}}_{1}-{\hat{Q}}_{2})\pm 1.96\sqrt{{\left(S{\hat{E}}_{1}\right)}^{2}+{\left(S{\hat{E}}_{2}\right)}^{2}}$$

where ${\hat{Q}}_{1}$ and ${\hat{Q}}_{2}$ were the effect estimates for the two categories, and *SÊ*_1_ and *SÊ*_2_ were their respective standard errors, respectively (22).

### Sensitivity Analysis

Air pollutants usually have a high interaction effect, so we built two-pollutant models for the sensitivity analysis. We changed the df of natural cubic spline function from 4 to 6 for temperature and adjusted the lag effect of temperature for single-day lags (from lag0 to lag6) and multi-day moving average lags (from lag01 to lag06) to explore the robustness of the associations between air pollutants and hospital admissions for childhood asthma.

All analyses were performed using the “gnm” and “splines” packages in R software (version 4.1.1). The results were expressed as the relative risk (RR) and 95% confidence interval (CI) of daily hospital admission for childhood asthma associated with per interquartile range (IQR) increase in PM_2.5_, PM_10_, SO_2_, NO_2_, CO, and O_3_. Statistical significance was defined as a two-tailed *p*-value < 0.05.

## Results

A total of 779 hospital admissions for childhood asthma between 1st January 2013 and 31st December 2016 from 56 hospitals in Beijing formed our study (Table 1). Most patients were male (65.3%), aged ≤ 6 years (65.5%), and in the warm season (66.1%). Table 2 shows the descriptive statistics of air pollutant concentrations and meteorological factors in our study. During the study, the daily mean concentrations of PM_2.5_, PM_10_, SO_2_, NO_2_, CO, and O_3_ were 80.94, 108.16, 17.07, 50.80 μg/m^3^, 1.27 mg/m^3^, and 71.37 μg/m^3^, respectively. The concentrations of SO_2_, NO_2_, and CO showed clear seasonal trends (i.e., higher in the cold seasons and lower in the warm seasons). However, the concentration of O_3_ showed an inverse trend (Supplementary Figure 1).

**Table 1:** Descriptive statistics of hospital admissions for childhood asthma in Beijing, China (2013–2016).

**Characteristics**	**Number of patients**	**Percentage (%)**
Total patients	779	-
Gender		
Male	509	65.3
Female	270	34.7
Age		
Preschool children	510	65.5
School-aged children	269	34.5
Season		
Cold season	264	33.9
Warm season	515	66.1

**Table 2:** Descriptive statistics of daily air pollutants concentrations and meteorological factors in Beijing, China (2013–2016).

	**Mean**	**SD**	**Min**	**P(25)**	**Median**	**P(75)**	**Max**	**IQR**
PM_2.5_ (μg/m^3^)	80.94	67.44	5.53	32.00	62.13	109.5	475.43	77.50
PM_10_ (μg/m^3^)	108.16	74.22	7.08	53.41	90.41	142.35	518.86	88.94
SO_2_ (μg/m^3^)	17.07	19.38	2.01	4.43	9.36	21.83	138.37	17.40
NO_2_ (μg/m^3^)	50.80	24.31	6.69	33.83	44.75	62.19	156.08	28.36
CO (mg/m^3^)	1.27	0.97	0.22	0.64	0.98	1.52	8.04	0.88
O_3_ (μg/m^3^)	71.37	55.33	2.18	30.78	59.85	94.93	331.08	64.15
Tmean (°C)	12.88	11.17	−16	2	14	23	32	-
RH (%)	53.43	19.86	8	38	53	69	97	-
WS(m/s)	9.29	4.75	3	6	8	11	34	-
AP (hPa)	1016.56	10.17	994	1008	1016	1025	1044	-

*SD, standard deviation; Min, minimum; Max, maximum; P(25), 25th percentile; P(75), 75th percentile; IQR, interquartile range; Tmean, daily mean temperature; RH, relative humidity; WS, wind speed; AP, air pressure*.

The Spearman's correlations between the different pollutants and meteorological factors are presented in Table 3. The daily concentrations of PM_2.5_, PM_10_, SO_2_, NO_2_, and CO were positively correlated with each other, especially the concentrations of PM_2.5_ and PM_10_ had a strong correlation (*r* = 0.91). In contrast, the concentration of O_3_ was negatively correlated with other pollutants. Moreover, the temperature was positively correlated with O_3_ and negatively correlated with SO_2_, NO_2_, and CO.

**Table 3:** Spearman's correlations between air pollutants and meteorological factors.

	**PM_**10**_**	**SO_**2**_**	**NO_**2**_**	**CO**	**O_**3**_**	**Tmean**	**RH**	**WS**	**AP**
PM_2.5_	0.91^*^	0.55^*^	0.73^*^	0.86^*^	−0.22^*^	−0.07^*^	0.48^*^	−0.57^*^	−0.02
PM_10_	-	0.58^*^	0.73^*^	0.74^*^	−0.20^*^	−0.05	0.29^*^	−0.49^*^	−0.06^*^
SO_2_	-	-	0.70^*^	0.67^*^	−0.45^*^	−0.56^*^	−0.22^*^	−0.20^*^	0.42^*^
NO_2_	-	-	-	0.79	−0.53^*^	−0.35^*^	0.21^*^	−0.62^*^	0.26^*^
CO	-	-	-	-	−0.47^*^	−0.31^*^	0.41^*^	−0.59^*^	0.21^*^
O_3_	-	-	-	-	-	0.70^*^	−0.06^*^	0.23^*^	−0.62^*^
Tmean	-	-	-	-	-	-	0.33^*^	−0.06^*^	−0.88^*^
RH	-	-	-	-	-	-	-	−0.59^*^	−0.29^*^
WS	-	-	-	-	-	-	-	-	0.04

* *indicates p value < 0.05; Tmean, daily mean temperature; RH, relative humidity; AP, air pressure; WS, wind speed*.

In the single-pollutant model (Table 4), higher levels of air pollutants except O_3_ were associated with increased risk of asthma hospital admission in children. The statistically significant positive associations with increased risk of asthma admission were observed in: PM_2.5_ at lag2; SO_2_ from lag2 to lag4 and lag03 to lag06; NO_2_ from lag3 to lag5 and lag04 to lag06. No significant association was found in PM_10_ and CO. The strongest effect was observed in NO_2_ at lag06 (*RR* = 1.25, 95%CI: 1.06–1.48), followed by SO_2_ at lag05 (*RR* = 1.17, 95%CI: 1.05–1.31). For O_3_, statistically significant negative associations were detected from lag0 to lag6 and lag01 to lag06, with the strongest effect at lag06 (*RR* = 0.74, 95%CI: 0.64–0.87).

**Table 4:** The relative risk (RR) of hospital admission for asthma in children per interquartile range (IQR) increase in different air pollutants in different lag days: single-pollutant model.

	**PM_**2.5**_**	**PM_**10**_**	**SO_**2**_**	**NO_**2**_**	**CO**	**O_**3**_**
	**RR (95%CI)**	**RR (95%CI)**	**RR (95%CI)**	**RR (95%CI)**	**RR (95%CI)**	**RR (95%CI)**
Lag0	0.93 (0.85, 1.02)	0.91 (0.83, 1.00)	1.00 (0.92, 1.09)	0.99 (0.90, 1.09)	0.92^*^ **(0.84, 1.00)**	0.84^*^ **(0.74, 0.95)**
Lag1	1.02 (0.93, 1.11)	1.00 (0.91, 1.10)	1.05 (0.97, 1.14)	1.04 (0.94, 1.14)	0.99 (0.91, 1.07)	0.81^*^ **(0.71, 0.91)**
Lag2	1.10^*^ **(1.01, 1.19)**	1.08 (0.99, 1.18)	1.11^*^ **(1.03, 1.20)**	1.09 (0.99, 1.20)	1.07 (0.99, 1.15)	0.85^*^ **(0.75, 0.95)**
Lag3	1.08 (0.99, 1.17)	1.09 (1.00, 1.19)	1.13^*^ **(1.05, 1.22)**	1.13^*^ **(1.03, 1.25)**	1.04 (0.97, 1.13)	0.86^*^ **(0.77, 0.97)**
Lag4	1.08 (0.99, 1.17)	1.06 (0.97, 1.16)	1.14^*^ **(1.06, 1.23)**	1.13^*^ **(1.03, 1.24)**	1.05 (0.97, 1.13)	0.81^*^ **(0.72, 0.91)**
Lag5	1.05 (0.97, 1.15)	1.04 (0.95, 1.14)	1.07 (0.98, 1.16)	1.11^*^ **(1.01, 1.23)**	1.02 (0.94, 1.10)	0.82^*^ **(0.73, 0.92)**
Lag6	1.05 (0.96, 1.14)	1.03 (0.94, 1.13)	1.05 (0.97, 1.14)	1.06 (0.96, 1.17)	1.00 (0.93, 1.09)	0.84^*^ **(0.75, 0.94)**
Lag01	0.97 (0.87, 1.07)	0.94 (0.84, 1.05)	1.03 (0.94, 1.14)	1.02 (0.91, 1.14)	0.94 (0.86, 1.03)	0.79^*^ **(0.69, 0.91)**
Lag02	1.03 (0.92, 1.15)	0.99 (0.88, 1.12)	1.08 (0.98, 1.19)	1.07 (0.94, 1.21)	0.99 (0.90, 1.09)	0.78^*^ **(0.68, 0.90)**
Lag03	1.06 (0.94, 1.20)	1.04 (0.91, 1.19)	1.12^*^ **(1.02, 1.24)**	1.13 (0.99, 1.29)	1.01 (0.91, 1.13)	0.78^*^ **(0.67, 0.90)**
Lag04	1.10 (0.96, 1.26)	1.07 (0.93, 1.24)	1.16^*^ **(1.05, 1.29)**	1.19^*^ **(1.03, 1.38)**	1.04 (0.92, 1.16)	0.76^*^ **(0.65, 0.88)**
Lag05	1.13 (0.97, 1.30)	1.10 (0.94, 1.28)	1.17^*^ **(1.05, 1.31)**	1.24^*^ **(1.06, 1.45)**	1.04 (0.92, 1.18)	0.75^*^ **(0.64, 0.87)**
Lag06	1.15 (0.98, 1.34)	1.11 (0.94, 1.31)	1.17^*^ **(1.04, 1.31)**	1.25^*^ **(1.06, 1.48)**	1.04 (0.91, 1.19)	0.74^*^ **(0.64, 0.87)**

* * and bold indicate p value < 0.05. The interquartile range (IQR) of daily concentrations of PM_2.5_, PM_10_, SO_2_, NO_2_, CO, and O_3_ were 77.50, 88.94, 17.40, 28.36 μg/m^3^, 0.88 mg/m^3^ and 64.15 μg/m^3^, respectively*.

According to the forementioned definition of strongest single lag day, the strongest single lag days were as follows: lag2 in PM_2.5_ (*RR* = 1.10, 95%CI: 1.01–1.19), lag3 in PM_10_ (*RR* = 1.10, 95%CI: 1.01–1.19), lag4 in SO_2_ (*RR* = 1.14, 95%CI: 1.06–1.23), lag3 in NO_2_ (*RR* = 1.13, 95%CI: 1.03–1.25), lag0 in CO (*RR* = 0.92,95%CI: 0.84–1.00) and lag1 in O_3_ (*RR* = 0.81, 95%CI: 0.71–0.91).

Tables 5–**7** present the association between air pollutants and childhood asthma hospital admissions stratified by gender, age, and season. Higher levels of PM_10_ and CO were significantly positively associated with increased risk of asthma admission in males [PM_10_ at lag2 (*RR* = 1.12, 95%CI: 1.01–1.25) and CO at lag2 (*RR* = 1.11, 95%CI: 1.02–1.20)] and in preschool children [PM_10_ from lag2 to lag3, with the maximum effect at lag2 (*RR* = 1.11, 95%CI: 1.00–1.23)]. However, among overall patients, higher levels of PM_10_ and CO were positively but insignificant associated with increased risk of asthma admission. More detailed results of stratification by gender, age, and season in multi-day moving-average lag models can be found in Supplementary Tables 1–3, respectively.

**Table 5:** The relative risk (RR) of hospital admission for asthma in children per interquartile range (IQR) increase in different air pollutants in different lag days: single-pollutant model, stratified by gender.

	**PM_**2.5**_**	**PM_**10**_**	**SO_**2**_**	**NO_**2**_**	**CO**	**O_**3**_**
	**RR (95%CI)**	**RR (95%CI)**	**RR (95%CI)**	**RR (95%CI)**	**RR (95%CI)**	**RR(95%CI)**
**Male**						
Lag0	0.91 (0.82, 1.02)	0.89 (0.79, 1.00)	1.00 (0.91, 1.11)	0.98 (0.87, 1.10)	0.90^*^ **(0.81, 0.99)**	0.83^*^ **(0.72, 0.97)**
Lag1	1.02 (0.92, 1.13)	0.99 (0.89, 1.11)	1.07 (0.97, 1.17)	1.05 (0.94, 1.17)	0.99 (0.90, 1.08)	0.82^*^ **(0.71, 0.95)**
Lag2	1.14^*^ **(1.04, 1.25)**	1.12^*^ **(1.01, 1.25)**	1.12^*^ **(1.03, 1.23)**	1.14^*^ **(1.02, 1.27)**	1.11^*^ **(1.02, 1.20)**	0.90 (0.79, 1.04)
Lag3	1.06 (0.96, 1.18)	1.10 (0.99, 1.22)	1.14^*^ **(1.04, 1.24)**	1.15^*^ **(1.03, 1.29)**	1.05 (0.96, 1.15)	0.90 (0.79, 1.03)
Lag4	1.09 (0.98, 1.20)	1.07 (0.96, 1.19)	1.14^*^ **(1.05, 1.25)**	1.14^*^ **(1.02, 1.27)**	1.06 (0.98, 1.16)	0.80^*^ **(0.69, 0.92)**
Lag5	1.08 (0.98, 1.19)	1.05 (0.94, 1.17)	1.10^*^ **(1.01, 1.20)**	1.14^*^ **(1.02, 1.27)**	1.04 (0.95, 1.14)	0.78^*^ **(0.68, 0.90)**
Lag6	1.06 (0.96, 1.18)	1.04 (0.94, 1.16)	1.07 (0.97, 1.17)	1.09 (0.98, 1.22)	1.02 (0.93, 1.12)	0.77^*^ **(0.67, 0.89)**
Lag06	1.18 (0.99, 1.42)	1.14 (0.95, 1.38)	1.20^*^ **(1.06, 1.37)**	1.32^*^ **(1.09, 1.61)**	1.08 (0.93, 1.26)	0.74^*^ **(0.62, 0.89)**
**Female**						
Lag0	0.98 (0.84, 1.14)	0.95 (0.80, 1.11)	1.00 (0.87, 1.16)	1.02 (0.87, 1.21)	0.96 (0.84, 1.11)	0.85 (0.69, 1.04)
Lag1	1.01 (0.87, 1.17)	1.01 (0.86, 1.18)	1.02 (0.88, 1.18)	1.02 (0.86, 1.20)	0.98 (0.86, 1.12)	0.78^*^ **(0.63, 0.96)**
Lag2	1.00 (0.86, 1.17)	0.99 (0.84, 1.16)	1.09 (0.95, 1.25)	0.99 (0.84, 1.17)	0.98 (0.85, 1.13)	0.75^*^ **(0.61, 0.92)**
Lag3	1.10 (0.95, 1.27)	1.07 (0.92, 1.25)	1.11 (0.97, 1.27)	1.09 (0.92, 1.28)	1.03 (0.90, 1.18)	0.79^*^ **(0.65, 0.97)**
Lag4	1.06 (0.91, 1.22)	1.05 (0.9, 1.22)	1.13 (0.99, 1.30)	1.11 (0.94, 1.31)	1.02 (0.89, 1.16)	0.83 (0.69, 1.01)
Lag5	1.00 (0.86, 1.16)	1.02 (0.87, 1.19)	0.99 (0.86, 1.15)	1.06 (0.90, 1.25)	0.96 (0.83, 1.11)	0.89 (0.74, 1.08)
Lag6	1.01 (0.87, 1.18)	1.00 (0.85, 1.18)	1.02 (0.88, 1.18)	1.00 (0.84, 1.19)	0.97 (0.84, 1.12)	0.95 (0.79, 1.14)
Lag06	1.07 (0.82, 1.41)	1.05 (0.79, 1.39)	1.10 (0.90, 1.34)	1.12 (0.84, 1.50)	0.95 (0.75, 1.21)	0.75^*^ **(0.58, 0.97)**

* * and bold indicate p value < 0.05. The interquartile range (IQR) of daily concentrations of PM_2.5_, PM_10_, SO_2_, NO_2_, CO, and O_3_ were 77.50, 88.94, 17.40, 28.36 μg/m^3^, 0.88 mg/m^3^ and 64.15 μg/m^3^, respectively*.

According to gender-stratified analysis (Table 5; Supplementary Table 1), for males, significant positive associations with increased risk of asthma admission were found in: PM_2.5_ at lag2; PM_10_ at lag2; SO_2_ from lag2 to lag 5, and from lag 03 to lag06; NO_2_ from lag2 to lag5, and from lag04 to lag06; CO at lag2. For females, higher levels of PM_2.5_, PM_10_, SO_2_, NO_2_, and CO were positively but insignificantly associated with increased risk of asthma admission. Both in males and females, higher level of O_3_ was significantly negatively associated with increased risk of asthma admission. Although the effects of all six pollutants (especially SO_2_ and NO_2_) in males seemed to be stronger than in females, we did not detect any significant differences between the two groups.

According to age-stratified analysis (Table 6; Supplementary Table 2), for preschool children, we only observed significant positive associations with increased risk of asthma admission in: PM_2.5_ at lag2; PM_10_ from lag2 to lag3; SO_2_ from lag2 to lag4 and lag02 to lag06; NO_2_ from lag3 to lag4 and lag04 to lag 06. Both in preschool children and school-aged children, higher level of O_3_ was significantly negatively associated with increased risk of asthma admission. No significant association was found in CO in all age groups. Except CO, the effects of pollutants in preschool children were stronger than in school-aged children, while significant differences were detected only in SO_2_ and O_3_ between the two groups.

**Table 6:** The relative risk (RR) of hospital admission for asthma in children per interquartile range (IQR) increase in different air pollutants in different lag days: single-pollutant model, stratified by age.

	**PM_**2.5**_**	**PM_**10**_**	**SO_**2**_**	**NO_**2**_**	**CO**	**O_**3**_**
	**RR (95%CI)**	**RR (95%CI)**	**RR (95%CI)**	**RR (95%CI)**	**RR (95%CI)**	**RR(95%CI)**
**Preschool children**						
Lag0	0.94 (0.84, 1.05)	0.93 (0.83, 1.05)	1.02 (0.92, 1.12)	0.99 (0.89, 1.11)	0.91 (0.82, 1.00)	0.81^*^ **(0.69, 0.95)**
Lag1	1.04 (0.94, 1.16)	1.04 (0.94, 1.16)	1.09 (1.00, 1.19)	1.06 (0.95, 1.19)	1.00 (0.92, 1.09)	0.77^*^ **(0.65, 0.90)**
Lag2	1.11^*^ **(1.01, 1.23)**	1.11^*^ **(1.00, 1.23)**	1.15^*^ **(1.05, 1.25)**	1.12 (1.00, 1.25)	1.08 (0.99, 1.17)	0.81^*^ **(0.70, 0.95)**
Lag3	1.08 (0.98, 1.20)	1.11^*^ **(1.00, 1.23)**	1.15^*^ (1.06, 1.25)	1.14^*^ **(1.02, 1.28)**	1.05 (0.96, 1.14)	0.83^*^ **(0.71, 0.96)**
Lag4	1.09 (0.99, 1.20)	1.10 (0.99, 1.22)	1.19^*^ (1.10, 1.29)	1.16^*^ **(1.04, 1.29)**	1.05 (0.96, 1.15)	0.73^*^ **(0.62, 0.86)**
Lag5	1.05 (0.95, 1.16)	1.05 (0.94, 1.17)	1.08 (0.99, 1.18)	1.10 (0.98, 1.23)	0.99 (0.91, 1.09)	0.75^*^ **(0.64, 0.88)**
Lag6	1.05 (0.95, 1.16)	1.04 (0.93, 1.16)	1.05 (0.96, 1.15)	1.03 (0.92, 1.15)	0.98 (0.89, 1.07)	0.78^*^ **(0.67, 0.91)**
Lag06	1.19 (0.99, 1.43)	1.20 (0.99, 1.45)	1.23^*^ **(1.08, 1.39)**	1.29^*^ **(1.06, 1.56)**	1.04 (0.89, 1.21)	0.67^*^ **(0.54, 0.82)**
**School-aged children**						
Lag0	0.92 (0.79, 1.08)	0.86 (0.72, 1.02)	0.96 (0.81, 1.14)	0.99 (0.83, 1.17)	0.94 (0.81, 1.10)	0.88 (0.74, 1.06)
Lag1	0.94 (0.80, 1.11)	0.88 (0.74, 1.05)	0.91 (0.76, 1.08)	0.97 (0.81, 1.15)	0.94 (0.81, 1.09)	0.87 (0.72, 1.04)
Lag2	1.05 (0.90, 1.22)	1.00 (0.85, 1.18)	0.99 (0.84, 1.16)	1.00 (0.84, 1.20)	1.03 (0.89, 1.18)	0.90 (0.76, 1.07)
Lag3	1.06 (0.91, 1.23)	1.05 (0.90, 1.23)	1.06 (0.90, 1.23)	1.10 (0.92, 1.31)	1.03 (0.90, 1.19)	0.92 (0.77, 1.08)
Lag4	1.04 (0.89, 1.21)	0.98 (0.83, 1.16)	0.98 (0.83, 1.15)	1.06 (0.89, 1.26)	1.04 (0.90, 1.19)	0.93 (0.79, 1.10)
Lag5	1.06 (0.91, 1.23)	1.02 (0.87, 1.20)	1.02 (0.88, 1.20)	1.15 (0.96, 1.36)	1.07 (0.93, 1.22)	0.93 (0.78, 1.09)
Lag6	1.03 (0.89, 1.20)	1.01 (0.86, 1.19)	1.05 (0.91, 1.23)	1.16 (0.97, 1.38)	1.07 (0.94, 1.23)	0.93 (0.79, 1.10)
Lag06	1.04 (0.79, 1.38)	0.92 (0.69, 1.23)	0.97 (0.77, 1.23)	1.17 (0.86, 1.58)	1.04 (0.81, 1.33)	0.87 (0.70, 1.08)

* * and bold indicate p value < 0.05. The interquartile range (IQR) of daily concentrations of PM_2.5_, PM_10_, SO_2_, NO_2_, CO, and O_3_ were 77.50, 88.94, 17.40, 28.36 μg/m^3^, 0.88 mg/m^3^ and 64.15 μg/m^3^, respectively*.

According to season-stratified analysis (Table 7; Supplementary Table 3), for the cold season, significant positive associations with increased risk of asthma admission were found in: SO_2_ from lag1 to lag6 and lag02 to lag06; NO_2_ at lag3. For the warm season, significant positive associations were found in: PM_2,5_ from lag05 to lag 06; NO_2_ from lag4 to lag6, at lag01 and from lag03 to lag06. Both in the cold season and the warm season, higher level of O_3_ was significantly negatively associated with increased risk of asthma admission. The effects of NO_2_ in the warm season were significantly stronger than in the cold season. While the effects of SO_2_ was significantly stronger in the cold season than in the warm season. The effects of PM_2.5_ seem to be stronger in the warm season, while no statistically significances were detected between two seasons. The maximum protective effects of O_3_ were found in the cold season, but it had no statistically significant differences compared with in the warm season.

**Table 7:** The relative risk (RR) of hospital admission for asthma in children per interquartile range (IQR) increase in different air pollutants in different lag days: single-pollutant model, stratified by season.

	**PM_**2.5**_**	**PM_**10**_**	**SO_**2**_**	**NO_**2**_**	**CO**	**O_**3**_**
	**RR (95%CI)**	**RR (95%CI)**	**RR (95%CI)**	**RR (95%CI)**	**RR (95%CI)**	**RR(95%CI)**
**Cold season**						
Lag0	0.89 (0.79, 1.01)	0.88 (0.77, 1.00)	1.04 (0.95, 1.15)	0.92 (0.81, 1.04)	0.89^*^ **(0.81, 0.99)**	0.73 (0.50, 1.07)
Lag1	0.98 (0.87, 1.10)	0.96 (0.84, 1.09)	1.10^*^ **(1.00, 1.20)**	0.98 (0.86, 1.11)	0.95 (0.86, 1.05)	0.71 (0.50, 1.02)
Lag2	1.11 (1.00, 1.24)	1.10 (0.98, 1.24)	1.17^*^ **(1.08, 1.28)**	1.10 (0.98, 1.24)	1.07 (0.98, 1.16)	0.80 (0.56, 1.12)
Lag3	1.09 (0.98, 1.22)	1.10 (0.98, 1.23)	1.20^*^ (1.00.1, 1.30)	1.13^*^ **(1.00, 1.27)**	1.05 (0.97, 1.15)	0.67^*^ **(0.47, 0.96)**
Lag4	1.07 (0.96, 1.20)	1.07 (0.95, 1.20)	1.19^*^ **(1.10, 1.29)**	1.10 (0.98, 1.24)	1.04 (0.95, 1.14)	0.67^*^ **(0.47, 0.96)**
Lag5	1.01 (0.90, 1.14)	1.02 (0.91, 1.15)	1.13^*^ **(1.04, 1.23)**	1.05 (0.93, 1.19)	0.99 (0.90, 1.09)	0.67^*^ **(0.47, 0.96)**
Lag6	1.00 (0.89, 1.13)	1.01 (0.89, 1.14)	1.12^*^ **(1.03, 1.22)**	1.02 (0.90, 1.15)	1.01 (0.92, 1.10)	0.76 (0.54, 1.08)
Lag06	1.09 (0.88, 1.35)	1.10 (0.88, 1.38)	1.32^*^ **(1.16, 1.49)**	1.16 (0.92, 1.45)	1.01 (0.86, 1.19)	0.55^*^ **(0.34, 0.90)**
**Warm season**						
Lag0	0.99 (0.86, 1.14)	0.95 (0.82, 1.11)	0.93 (0.77, 1.14)	1.15 (0.98, 1.35)	1.00 (0.86, 1.17)	0.83^*^ **(0.73, 0.96)**
Lag1	1.08 (0.95, 1.24)	1.07 (0.93, 1.24)	0.97 (0.80, 1.18)	1.16 (0.99, 1.36)	1.10 (0.96, 1.27)	0.81^*^ **(0.71, 0.93)**
Lag2	1.10 (0.96, 1.25)	1.07 (0.93, 1.23)	1.04 (0.86, 1.25)	1.09 (0.92, 1.28)	1.10 (0.96, 1.27)	0.86^*^ **(0.76, 0.98)**
Lag3	1.08 (0.94, 1.23)	1.10 (0.96, 1.27)	1.01 (0.84, 1.22)	1.15 (0.98, 1.35)	1.05 (0.91, 1.22)	0.90 (0.79, 1.01)
Lag4	1.12 (0.98, 1.28)	1.10 (0.95, 1.26)	1.14 (0.96, 1.36)	1.20^*^ **(1.02, 1.41)**	1.13 (0.98, 1.29)	0.85^*^ **(0.75, 0.97)**
Lag5	1.13 (0.99, 1.29)	1.13 (0.99, 1.30)	0.97 (0.80, 1.17)	1.22^*^ **(1.03, 1.43)**	1.11 (0.96, 1.27)	0.86^*^ **(0.76, 0.97)**
Lag6	1.12 (0.98, 1.28)	1.10 (0.96, 1.27)	0.99 (0.82, 1.20)	1.14 (0.96, 1.34)	1.04 (0.89, 1.21)	0.87^*^ **(0.77, 0.98)**
Lag06	1.34^*^ **(1.05, 1.71)**	1.25 (0.98, 1.59)	1.06 (0.81, 1.40)	1.47^*^ **(1.13, 1.91)**	1.26 (0.98, 1.62)	0.78^*^ **(0.66, 0.92)**

* * and bold indicate p value < 0.05. The interquartile range (IQR) of daily concentrations of PM_2.5_, PM_10_, SO_2_, NO_2_, CO, and O_3_ were 77.50, 88.94, 17.40, 28.36 μg/m^3^, 0.88 mg/m^3^ and 64.15 μg/m^3^, respectively*.

The results of the two-pollutants models are presented in Table 8. Strongest single lag days for each pollutant were determined in the single-pollutant model, and then we added additional air pollutants for adjustment. Because the concentrations of PM_2.5_ and PM_10_ were strongly correlated, we analyzed all pollutants except for PM_10_ in two-pollutant models. The effects of SO_2_, NO_2_, and O_3_ were still robust and significant in two-pollutant models. However, the estimate of PM_2.5_ became not statistically significant after adjustment for O_3_, and the estimate of CO became not statistically significant after adjustment for PM_2.5_.

**Table 8:** The relative risk (RR) of hospital admission for asthma in children per interquartile range (IQR) increase in different air pollutants in different strongest single lag days: two-pollutant model.

	**Unadjusted**	**+PM_**2.5**_**	**+SO_**2**_**	**+NO_**2**_**	**+CO**	**+O_**3**_**
	**RR (95%CI)**	**RR (95%CI)**	**RR (95%CI)**	**RR (95%CI)**	**RR (95%CI)**	**RR (95%CI)**
PM_2.5_	1.10^*^ **(1.01, 1.19)**	-	1.10^*^ **(1.01, 1.19)**	1.10^*^ **(1.01, 1.19)**	1.11^*^ **(1.02, 1.20)**	1.08 (0.99, 1.18)
SO_2_	1.14^*^ **(1.06, 1.23)**	1.14^*^ **(1.06, 1.23)**	-	1.14^*^ **(1.06, 1.23)**	1.14^*^ **(1.06, 1.23)**	1.13^*^ **(1.05, 1.22)**
NO_2_	1.13^*^ **(1.03, 1.25)**	1.14^*^ **(1.03, 1.25)**	1.13^*^ **(1.03, 1.25)**	-	1.14^*^ **(1.03, 1.25)**	1.11^*^ **(1.01, 1.23)**
CO	0.92^*^ **(0.84, 1.00)**	0.90 (0.77, 1.05)	0.87^*^ **(0.78, 0.97)**	0.79^*^ **(0.69, 0.92)**	-	0.88^*^ **(0.81, 0.97)**
O_3_	0.81^*^ **(0.71, 0.91)**	0.80^*^ **(0.71, 0.91)**	0.81^*^ **(0.71, 0.91)**	0.80^*^ **(0.71, 0.91)**	0.79^*^ **(0.69, 0.89)**	-

* * and bold indicate p value < 0.05*.

*The interquartile range (IQR) of daily concentrations of PM_2.5_, SO_2_, NO_2_, CO, and O_3_ were 77.50, 17.40, 28.36 μg/m^3^, 0.88 mg/m^3^ and 64.15 μg/m^3^, respectively*.

*The strongest single lag days of PM_2.5_, SO_2_, NO_2_, CO, and O_3_ was lag2, lag4, lag3, lag0, and lag1, respectively*.

Supplementary Tables 4, 5 show the RRs of asthma hospital admission per IQR increase in different pollutants from lag0 to lag6 and lag01 to lag06 after adjusting the lag effects of temperature and using different dfs of nature cubic spline, respectively. The estimates of PM_2.5_, SO_2_, NO_2_, and O_3_ were robust and remained statistically significant. The estimates of CO at lag2 became significant after adjusting the lag effect of temperature, while the estimate of CO at lag0 became not statistically significant after adjustment for the lag effects of temperature or using different dfs of nature cubic spline.

## Discussion

To the best of our knowledge, this is the first study on the association between short-term exposure to six air pollutants and asthma hospital admissions in children covering all districts in Beijing, a mid-high latitude region with serious air pollution and high-quality healthcare at the same time. By employing a time-stratified case-crossover design, we observed exposure to air pollutants (PM_2.5_, NO_2_, and SO_2_) is associated with increased risk of hospital admission for asthma in children. In stratified analyses, preschool children (0–6 years) were more susceptible to SO_2_ than school-aged children (7–18 years old). SO_2_ showed a stronger adverse effect in the cold season, and NO_2_ showed a stronger adverse effect in the warm season. Conversely, O_3_ showed a protective effect in our study.

Particulate matter can deposit in the airway and directly elicit airway inflammation, mucosal edema, and cytotoxicity (23). Our study found that PM_2.5_ and PM_10_ had adverse effects on daily childhood asthma hospital admissions. Previous studies also showed that PM_2.5_ and PM_10_ had adverse effects on asthma development and attacks in children (8, 24). In this study, the level of PM_10_ was only significantly associated with childhood asthma admissions in males and preschool children, and the effects of PM_2.5_ were stronger than PM_10_, which was consistent with previous studies (8). Compared with PM_10_, the smaller size of PM_2.5_ may lead to a greater possibility of reaching the small airways and the alveoli of the lung, and greater absorption of allergens (25), thus induced the stronger effect.

SO_2_ is a well-known inhalable air pollutant, which can be a potential trigger factor for asthma exacerbation. In our study, SO_2_ showed a persistently strong effect on childhood asthma, which remained significant after adjusting the effects of meteorological factors and other pollutants. Previous studies also found a positive association between SO_2_ and childhood hospital admissions (8, 9). SO_2_ can exacerbate asthma by giving rise to airway inflammation and local oxidative stress (26, 27). Compared with non-asthmatic subjects, subjects with asthma had greater susceptibility to the effects of SO_2_ and were prone to developing bronchoconstriction after exposure to lower concentrations of SO_2_ (28, 29). These might explain the positive correlation between SO_2_ and asthma hospital admissions detected in our study.

NO_2_ is mainly generated from fossil fuel combustion and has been proved as a risk factor of asthma, which causes a substantial global burden of disease (30). Our study found that NO_2_ had a strong adverse effect on childhood asthma, which was consistent with previous studies (8, 31, 32). It has been demonstrated that NO_2_ could cause airway inflammation in healthy individuals (33) and enhance the airway response to inhaled allergen in asthmatics (34–36), which could be the potential mechanism of causing asthma development and attacks.

The existing evidence on the associations between O_3_ exposure and childhood asthma is controversial. A study in Taiwan, China (37) and our study found that O_3_ had a protective effect on children with asthma, which was opposite to the results in some other studies (31, 38–40). O_3_ can impact pediatric asthma in two opposite ways. O_3_ could cause asthma exacerbation by evoking oxidative stress and airway inflammation and increasing the reactivity to allergens in asthmatics (41). On the other hand, O_3_ shows a protective effect by protecting lungs from some respiratory viral infections (42). This could be a possible explanation of the protective effect of O_3_ revealed in this study. Additionally, the protective effect of O_3_ might be a result of the negative correlation of O_3_ with temperature and other pollutants, which could be a probable explanation of the maximum protective effect of O_3_ in the cold season in our study.

The incomplete combustion of fossil fuel can produce CO, which could successfully compete with oxygen in binding to hemoglobin and result in tissue hypoxia. In our study, CO showed a significant protective effect in lag0, which was not robust and might be caused by misclassification of using admission date as the date of an asthma attack. On other lag days, we only found a significant positive association between CO and increased risk of asthma admission in males at lag2. The weak effect of CO might attribute to the low concentration of CO in Beijing. The evidence on CO and asthma is limited. Several systematic reviews and meta-analysis studies showed that the concentration of CO was positively associated with higher prevalence and hospital utilization of asthma in children (7–9). There was a significant association between CO and childhood asthma hospital visits in a study in Shanghai (40). Further studies required to explore the effect of CO on childhood asthma.

In the stratified analyses, the associations between air pollution and asthma hospital admissions varied significantly by age, but no significant gender-specific differences were observed. Preschool children (0–6 years old) were more susceptible to SO_2_. Studies in Shijiazhuang (43), Chongqing (20), and Taiwan, China (37) found that air pollutants (PM_2.5_, SO_2_, and NO_2_) showed stronger effects in young children (0–5 years) as well. It is generally recognized that immature lungs, higher breathing rate (44), and predominantly oral breathing characteristics (45) are attributed to the high vulnerability among young children. Conversely, a study in Hefei found that school-aged children (6–18 years) were more susceptible to NO_2_ (31), which could be plausibly explained by more exposure to traffic-related air pollutants such as NO_2_ in school-aged children. For gender-specific results, there is a lack of consistent results as well (31, 32, 37). Some of the inconsistencies between those studies could be attributed to distinct regions, pollution characteristics, and unadjusted potential confounders.

We found that the effect of SO_2_ was stronger in the cold season, while the effect of NO_2_ was stronger in the warm season. Previous studies have shown inconsistent seasonal patterns of ambient air pollutant effects (20, 39, 46–49), which could be related to the temporal-spatial variation of pollutants and different patterns of activity in different seasons and regions. In the region Beijing locate in, fossil fuel combustion are the main sources of SO_2_ and NO_2_. Burning of solid fuels especially bulk coal is an important contributor to SO_2_ emissions, which could significantly increase the concentration of SO_2_ during winter (50). And NO_2_ emissions were mainly contributed by Diesel and gasoline combustion (51). The seasonal variability of air pollutants concentrations and sources could explain part of the seasonal differences in SO_2_ and NO_2_ in our study.

The study has several strengths. Firstly, we applied a time-stratified case-crossover design to minimize the effects of long-term and seasonal trends and control the influence of time-invariant confounders, including genetics, secondhand smoke, nutrition, socioeconomic status, and so on. Secondly, as many as six kinds of air pollutants and all districts of Beijing were enrolled in our study. Since Beijing is a special city with serious air pollution and high-quality healthcare, our study emphasizes the importance of reducing air pollution from a new perspective. Thirdly, we considered the interaction effects between different air pollutants and the lag effect of meteorological factors, which were comprehensively adjusted in our analysis to accurately determine associations between each pollutant and childhood asthma hospital admissions. Additionally, stratified analyses were performed by gender, age, and season, which is essential for analyzing air pollution and asthma precisely.

The limitations of this study should be acknowledged. Firstly, similar to other ecological studies, we used city-wide average air pollutant concentrations from multiple fixed monitoring stations rather than personal exposure measures, which might not precisely represent the personal exposure dosage and caused exposure misclassification (52, 53). Secondly, although we applied a case-crossover design to control time-invariant confounders, it is still possible that unmeasured time-varying confounders contributed to our results. Finally, data from only one city were used in this study, which may limit the generalizability of our results. Multi-city analyses are required for further investigation on these pollutants.

## Conclusions

Our study assessed the effects of six air pollutants on childhood asthma hospital admissions in Beijing, a developed city with serious air condition in Northern China. This study observed adverse effects of PM_2.5_, SO_2_, and NO_2_ on hospital admissions for childhood asthma. Preschool children (0–6 years) were more vulnerable to SO_2_. These results suggested that high levels of air pollution had an adverse effect on childhood asthma even in a region with high-quality medical care. Therefore, it will be of great significance for governments to decrease childhood asthma admissions by controlling outdoor air pollution emission, and both particulate matter and gaseous pollutants should be concerned. Caregivers of children with asthma need to realize the seriousness of air pollution and reduce exposure to air pollution by taking protective measures, such as wearing anti-dust respirators or reducing outdoor activities when outdoor air quality is poor.

## Data Availability Statement

The government policy regulates disclosure of the datasets concerning this study. Requests to access these datasets should be directed to ZF (fanzhongjie@pumch.cn).

## Ethics Statement

The studies involving human participants were reviewed and approved by Peiking Union Medical College Hospital (PUMCH) Institutional Review Board. Written informed consent from the participants' legal guardian/next of kin was not required to participate in this study in accordance with the national legislation and the institutional requirements.

## Author Contributions

ZF obtained the original data and funding. ZF and YZhao designed the study. DK, XL, JF, and YZhang pre-processed the data. YZhao analyzed the data and drafted the manuscript. All authors contributed to the interpretation of data and revised the article. All authors read and approved the submitted version.

## Funding

This research was supported by Chinese Academy of Medical Sciences (CAMS) Initiative for Innovative Medicine [grant number 2017-I2M-2-001], National Natural Science Foundation [grant number 91643208], and National Key Research and Development Plan [grant numbers 2017YFC0211703 and 2015CB553402].

## Conflict of Interest

The authors declare that the research was conducted in the absence of any commercial or financial relationships that could be construed as a potential conflict of interest.

## Publisher's Note

All claims expressed in this article are solely those of the authors and do not necessarily represent those of their affiliated organizations, or those of the publisher, the editors and the reviewers. Any product that may be evaluated in this article, or claim that may be made by its manufacturer, is not guaranteed or endorsed by the publisher.

## References

[1] GBD 2016 Disease and Injury Incidence and Prevalence Collaborators. Global, regional, and national incidence, prevalence, and years lived with disability for 354 diseases and injuries for 195 countries and territories, 1990-2017: a systematic analysis for the Global Burden of Disease Study 2017. Lancet. (2018) 392:1789–858. 10.1016/S0140-6736(18)32279-7 30496104 PMC6227754

[2] Collaborators GBDCoD. Global, regional, and national age-sex-specific mortality for 282 causes of death in 195 countries and territories, 1980-2017: a systematic analysis for the Global Burden of Disease Study 2017. Lancet. (2018) 392:1736–88. 10.1016/S0140-6736(18)32203-7 30496103 PMC6227606

[3] GBD 2017 DALYs and HALE Collaborator. Global, regional, and national disability-adjusted life-years (DALYs) for 359 diseases and injuries and healthy life expectancy (HALE) for 195 countries and territories, 1990-2017: a systematic analysis for the Global Burden of Disease Study 2017. Lancet. (2018) 392:1859–922. 10.1016/S0140-6736(18)32335-3 30415748 PMC6252083

[4] Global Burden of disease. 2019. Available online at: https://vizhub.healthdata.org/gbd-compare/ (accessed January 29, 2021).

[5] Botturi K Langelot M Lair D Pipet A Pain M Chesne J. Preventing asthma exacerbations: what are the targets? Pharmacol Ther. (2011) 131:114–29. 10.1016/j.pharmthera.2011.03.010 21440000

[6] Forno E Celedon JC. Predicting asthma exacerbations in children. Curr Opin Pulm Med. (2012) 18:63–9. 10.1097/MCP.0b013e32834db288 22081091 PMC3296525

[7] Gasana J Dillikar D Mendy A Forno E Ramos Vieira E. Motor vehicle air pollution and asthma in children: a meta-analysis. Environ Res. (2012) 117:36–45. 10.1016/j.envres.2012.05.001 22683007

[8] Orellano P Quaranta N Reynoso J Balbi B Vasquez J. Effect of outdoor air pollution on asthma exacerbations in children and adults: Systematic review and multilevel meta-analysis. PLoS ONE. (2017) 12:e0174050. 10.1371/journal.pone.0174050 28319180 PMC5358780

[9] Zhang S Li G Tian L Guo Q Pan X. Short-term exposure to air pollution and morbidity of COPD and asthma in East Asian area: a systematic review and meta-analysis. Environ Res. (2016) 148:15–23. 10.1016/j.envres.2016.03.008 26995350

[10] Wang WQ Lin JT Zhou X Wang CZ Huang M Cai SX. Evaluation of asthma management from the surveys in 30 provinces of China in 2015-2016. Zhonghua Nei Ke Za Zhi. (2018) 57:15–20. 10.3760/cma.j.issn.0578-1426.2018.01.003 29325305

[11] Su N Lin J Chen P Li J Wu C Yin K. Evaluation of asthma control and patient's perception of asthma: findings and analysis of a nationwide questionnaire-based survey in China. J Asthma. (2013) 50:861–70. 10.3109/02770903.2013.808346 23713625

[12] Papadopoulos NG Christodoulou I Rohde G Agache I Almqvist C Bruno A. Viruses and bacteria in acute asthma exacerbations–a GA(2) LEN-DARE systematic review. Allergy. (2011) 66:458–68. 10.1111/j.1398-9995.2010.02505.x 21087215 PMC7159474

[13] Glezen WP. Asthma, influenza, and vaccination. J Allergy Clin Immunol. (2006) 118:1199–206. 10.1016/j.jaci.2006.08.032 17157648

[14] Cowling BJ Wong IO Ho LM Riley S Leung GM. Methods for monitoring influenza surveillance data. Int J Epidemiol. (2006) 35:1314–21. 10.1093/ije/dyl162 16926216

[15] Maclure M. The case-crossover design: a method for studying transient effects on the risk of acute events. Am J Epidemiol. (1991) 133:144–53. 10.1093/oxfordjournals.aje.a115853 1985444

[16] Carracedo-Martinez E Taracido M Tobias A Saez M Figueiras A. Case-crossover analysis of air pollution health effects: a systematic review of methodology and application. Environ Health Perspect. (2010) 118:1173–82. 10.1289/ehp.0901485 20356818 PMC2920078

[17] Jaakkola JJ. Case-crossover design in air pollution epidemiology. Eur Respir J Suppl. (2003) 40:81s–5s. 10.1183/09031936.03.00402703 12762580

[18] Armstrong BG Gasparrini A Tobias A. Conditional poisson models: a flexible alternative to conditional logistic case cross-over analysis. BMC Med Res Methodol. (2014) 14:122. 10.1186/1471-2288-14-122 25417555 PMC4280686

[19] Figueiras A Cadarso-Suarez C. Application of nonparametric models for calculating odds ratios and their confidence intervals for continuous exposures. Am J Epidemiol. (2001) 154:264–75. 10.1093/aje/154.3.264 11479192

[20] Ding L Zhu D Peng D Zhao Y. Air pollution and asthma attacks in children: a case-crossover analysis in the city of Chongqing, China. Environ Pollut. (2017) 220(Pt A):348–53. 10.1016/j.envpol.2016.09.070 27692885

[21] Zhang Y Ding Z Xiang Q Wang W Huang L Mao F. Short-term effects of ambient PM1 and PM25 air pollution on hospital admission for respiratory diseases: Case-crossover evidence from Shenzhen, China. Int J Hyg Environ Health. (2020) 224:113418. 10.1016/j.ijheh.2019.11.001 31753527

[22] Chen R Kan H Chen B Huang W Bai Z Song G. Association of particulate air pollution with daily mortality: the China air pollution and health effects study. Am J Epidemiol. (2012) 175:1173–81. 10.1093/aje/kwr425 22510278

[23] Henderson RF Benson JM Hahn FF Hobbs CH Jones RK Mauderly JL. New approaches for the evaluation of pulmonary toxicity: bronchoalveolar lavage fluid analysis. Fundam Appl Toxicol. (1985) 5:451–8. 10.1016/0272-0590(85)90092-2 3891479

[24] Khreis H Kelly C Tate J Parslow R Lucas K Nieuwenhuijsen M. Exposure to traffic-related air pollution and risk of development of childhood asthma: a systematic review and meta-analysis. Environ Int. (2017) 100:1–31. 10.1016/j.envint.2016.11.012 27881237

[25] Kim KH Kabir E Kabir S. A review on the human health impact of airborne particulate matter. Environ Int. (2015) 74:136–43. 10.1016/j.envint.2014.10.005 25454230

[26] Reno AL Brooks EG Ameredes BT. Mechanisms of heightened airway sensitivity and responses to inhaled SO2 in asthmatics. Environ Health Insights. (2015) 9:13–25. 25922579 10.4137/EHI.S15671 PMC4384764

[27] Cai C Xu J Zhang M Chen XD Li L Wu J. Prior SO2 exposure promotes airway inflammation and subepithelial fibrosis following repeated ovalbumin challenge. Clin Exp Allergy. (2008) 38:1680–7. 10.1111/j.1365-2222.2008.03053.x 18631350

[28] Sheppard D Wong WS Uehara CF Nadel JA Boushey HA. Lower threshold and greater bronchomotor responsiveness of asthmatic subjects to sulfur dioxide. Am Rev Respir Dis. (1980) 122:873–8. 10.1164/arrd.1980.122.6.873 7458061

[29] Linn WS Avol EL Peng RC Shamoo DA Hackney JD. Replicated dose-response study of sulfur dioxide effects in normal, atopic, and asthmatic volunteers. Am Rev Respir Dis. (1987) 136:1127–34. 10.1164/ajrccm/136.5.1127 3674575

[30] Achakulwisut P Brauer M Hystad P Anenberg SC. Global, national, and urban burdens of paediatric asthma incidence attributable to ambient NO2 pollution: estimates from global datasets. Lancet Planet Health. (2019) 3:e166–e78. 10.1016/S2542-5196(19)30046-4 30981709

[31] Zhang Y Ni H Bai L Cheng Q Zhang H Wang S. The short-term association between air pollution and childhood asthma hospital admissions in urban areas of Hefei City in China: a time-series study. Environ Res. (2019) 169:510–6. 10.1016/j.envres.2018.11.043 30544078

[32] Iskandar A Andersen ZJ Bonnelykke K Ellermann T Andersen KK Bisgaard H. Coarse and fine particles but not ultrafine particles in urban air trigger hospital admission for asthma in children. Thorax. (2012) 67:252–7. 10.1136/thoraxjnl-2011-200324 22156960

[33] Sandström T Stjernberg N Eklund A Ledin MC Bjermer L Kolmodin-Hedman B. Inflammatory cell response in bronchoalveolar lavage fluid after nitrogen dioxide exposure of healthy subjects: a dose-response study. Eur Respir J. (1991) 4:332–9. 1864348

[34] Devalia JL Rusznak C Herdman MJ Trigg CJ Tarraf H Davies RJ. Effect of nitrogen dioxide and sulphur dioxide on airway response of mild asthmatic patients to allergen inhalation. Lancet. (1994) 344:1668–71. 10.1016/S0140-6736(94)90458-8 7996960

[35] Tunnicliffe WS Burge PS Ayres JG. Effect of domestic concentrations of nitrogen dioxide on airway responses to inhaled allergen in asthmatic patients. Lancet. (1994) 344:1733–6. 10.1016/S0140-6736(94)92886-X 7997002

[36] Strand V Svartengren M Rak S Barck C Bylin G. Repeated exposure to an ambient level of NO2 enhances asthmatic response to a nonsymptomatic allergen dose. Eur Respir J. (1998) 12:6–12. 10.1183/09031936.98.12010006 9701406

[37] Kuo CY Chan CK Wu CY Phan DV Chan CL. The short-term effects of ambient air pollutants on childhood asthma hospitalization in Taiwan: a national study. Int J Environ Res Public Health. (2019) 16:203. 10.3390/ijerph16020203 30642061 PMC6351918

[38] Ko FW Tam W Wong TW Lai CK Wong GW Leung TF. Effects of air pollution on asthma hospitalization rates in different age groups in Hong Kong. Clin Exp Allergy. (2007) 37:1312–9. 10.1111/j.1365-2222.2007.02791.x 17845411

[39] Zheng XY Ding H Jiang LN Chen SW Zheng JP Qiu M. Association between air pollutants and asthma emergency room visits and hospital admissions in time series studies: a systematic review and meta-analysis. PLoS ONE. (2015) 10:e0138146. 10.1371/journal.pone.0138146 26382947 PMC4575194

[40] Liu L Liu C Chen R Zhou Y Meng X Hong J. Associations of short-term exposure to air pollution and emergency department visits for pediatric asthma in Shanghai, China. Chemosphere. (2021) 263:127856. 10.1016/j.chemosphere.2020.127856 32822929

[41] Kehrl HR Peden DB Ball B Folinsbee LJ. Horstman D. Increased specific airway reactivity of persons with mild allergic asthma after 76 hours of exposure to 016 ppm ozone. J Allergy Clin Immunol. (1999) 104:1198–204. 10.1016/S0091-6749(99)70013-8 10589001

[42] Wolcott JA Zee YC Osebold JW. Exposure to ozone reduces influenza disease severity and alters distribution of influenza viral antigens in murine lungs. Appl Environ Microbiol. (1982) 44:723–31. 10.1128/aem.44.3.723-731.1982 6182839 PMC242082

[43] Fu GQ Jiang YF Liu LP Liu HY Zhou J Cui XW. Effects of PM25 exposure in different air quality grades on daily outpatient visits for childhood asthma in Shijiazhuang, China. Biomed Environ Sci. (2018) 31:888–92. 10.3967/bes2018.120 30636659

[44] Sigmund E De Ste Croix M Miklankova L Fromel K. Physical activity patterns of kindergarten children in comparison to teenagers and young adults. Eur J Public Health. (2007) 17:646–51. 10.1093/eurpub/ckm033 17434876

[45] Esposito S Tenconi R Lelii M Preti V Nazzari E Consolo S. Possible molecular mechanisms linking air pollution and asthma in children. BMC Pulm Med. (2014) 14:31. 10.1186/1471-2466-14-31 24581224 PMC3941253

[46] Kuo CY Pan RH Chan CK Wu CY Phan DV Chan CL. Application of a time-stratified case-crossover design to explore the effects of air pollution and season on childhood asthma hospitalization in cities of differing urban patterns: big data analytics of government open data. Int J Environ Res Public Health. (2018) 15:647. 10.3390/ijerph15040647 29614737 PMC5923689

[47] Chen K Glonek G Hansen A Williams S Tuke J Salter A. The effects of air pollution on asthma hospital admissions in Adelaide, South Australia, 2003-2013: time-series and case-crossover analyses. Clin Exp Allergy. (2016) 46:1416–30. 10.1111/cea.12795 27513706

[48] Chang Q Liu S Chen Z Zu B Zhang H. Association between air pollutants and outpatient and emergency hospital visits for childhood asthma in Shenyang city of China. Int J Biometeorol. (2020) 64:1539–48. 10.1007/s00484-020-01934-9 32388688

[49] Ma Y Yue L Liu J He X Li L Niu J. Association of air pollution with outpatient visits for respiratory diseases of children in an ex-heavily polluted Northwestern city, China. BMC Public Health. (2020) 20:816. 10.1186/s12889-020-08933-w 32487068 PMC7265648

[50] Cheng NL Zhang DW Li YT Chen T Li JX Dong X. Analysis about spatial and temporal distribution of SO_2_ and an ambient SO_2_ pollution process in Beijing during 2000-2014. Huan jing ke xue. (2015) 36:3961–71. 26910979

[51] Xue Y Wu T Cui Y Gong B Li X Qin X. Energy consumption and pollutant emission of diesel-fired combustion from 2009 to 2018 in Beijing, China. J Environ Manag. (2021) 285:112137. 10.1016/j.jenvman.2021.112137 33588167

[52] Goldman GT Mulholland JA Russell AG Strickland MJ Klein M Waller LA. Impact of exposure measurement error in air pollution epidemiology: effect of error type in time-series studies. Environ Health. (2011) 10:61. 10.1186/1476-069X-10-61 21696612 PMC3146396

[53] Zeger SL Thomas D Dominici F Samet JM Schwartz J Dockery D. Exposure measurement error in time-series studies of air pollution: concepts and consequences. Environ Health Perspect. (2000) 108:419–26. 10.1289/ehp.00108419 10811568 PMC1638034

