# Supplementary material for: Increased Risk of Hospital Admission for Asthma in Children From Short-Term Exposure to Air Pollution: Case-Crossover Evidence From Northern China
Source: Front Public Health. 2021 Dec 17;9:798746. doi: 10.3389/fpubh.2021.798746 (PMC8718688; doi:10.3389/fpubh.2021.798746)
Supplement: Supplementary file 1 [file Data_Sheet_1.docx]

Supplementary Material

# Supplementary Figures and Tables

## Supplementary Figures


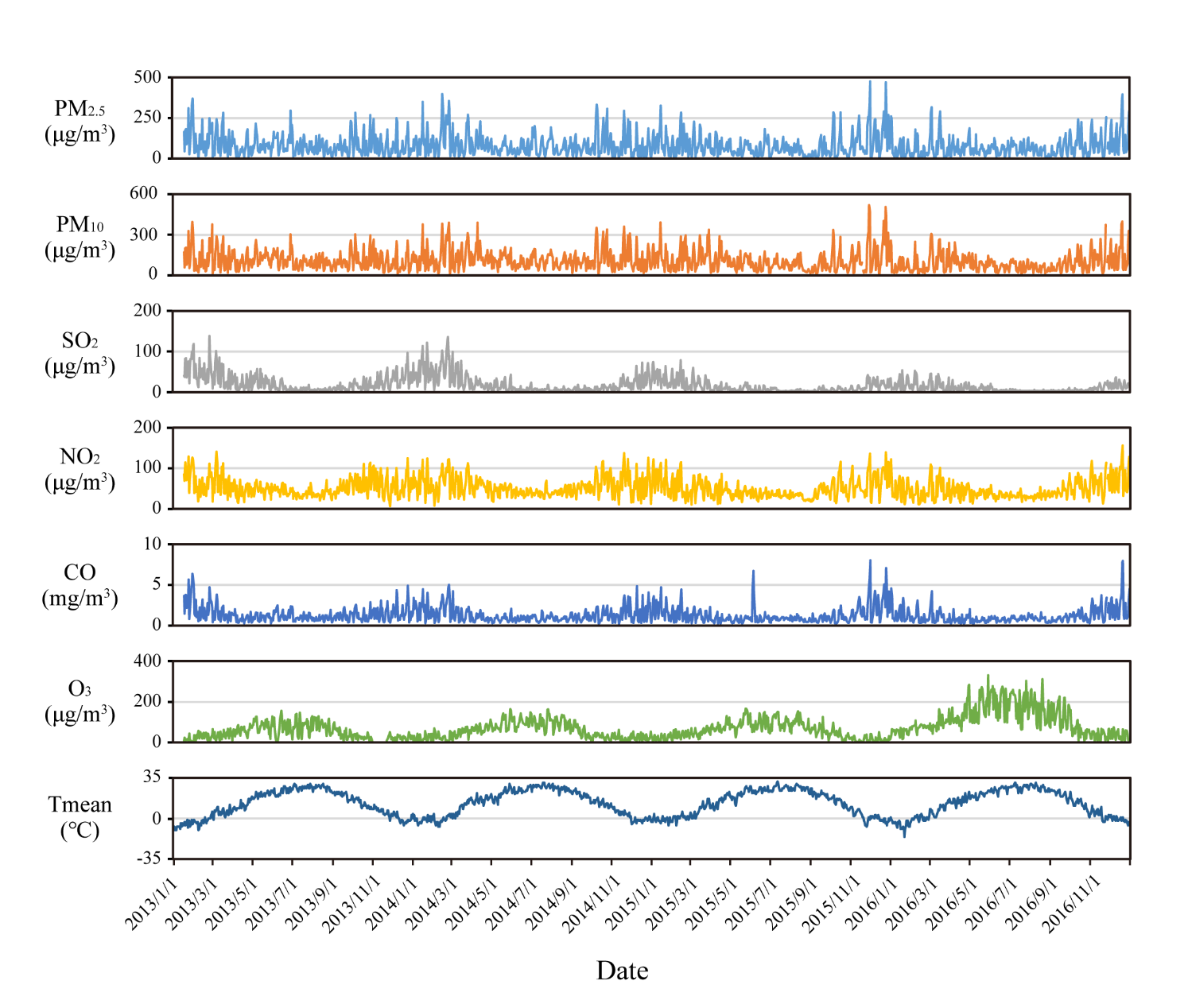


**Supplementary Figure 1.** Time series plots of daily concentrations of air pollutants and daily mean temperature in Beijing, China (2013-2016). Notes: Tmean, daily mean temperature.

## Supplementary Tables

**Supplementary Table 1.** The relative risk (RR) of hospital admission for asthma in children per interquartile range (IQR) increase in different air pollutants in different moving-average lags: single-pollutant model, stratified by gender

|  | **PM_2.5_** | **PM_10_** | **SO_2_** | **NO_2_** | **CO** | **O_3_** |
| --- | --- | --- | --- | --- | --- | --- |
|  | **RR (95%CI)** | **RR (95%CI)** | **RR (95%CI)** | **RR (95%CI)** | **RR (95%CI)** | **RR(95%CI)** |
| Male | | | | | | |
| Lag01 | 0.96  (0.85,1.08) | 0.93  (0.81,1.06) | 1.05  (0.94,1.16) | 1.02  (0.89,1.16) | 0.93  (0.84,1.04) | **0.80***  **(0.68,0.94)** |
| Lag02 | 1.04  (0.91,1.19) | 1.00  (0.87,1.15) | 1.10  (0.98,1.22) | 1.09  (0.94,1.26) | 1.00  (0.90,1.12) | **0.81***  **(0.69,0.96)** |
| Lag03 | 1.07  (0.93,1.24) | 1.06  (0.91,1.23) | **1.14***  **(1.01,1.28)** | 1.16  (0.99,1.36) | 1.03  (0.91,1.16) | **0.82***  **(0.69,0.97)** |
| Lag04 | 1.12  (0.95,1.30) | 1.09  (0.93,1.29) | **1.18***  **(1.05,1.33)** | **1.22***  **(1.03,1.45)** | 1.06  (0.93,1.21) | **0.79***  **(0.66,0.94)** |
| Lag05 | 1.15  (0.97,1.37) | 1.12  (0.94,1.34) | **1.20***  **(1.06,1.36)** | **1.28***  **(1.07,1.54)** | 1.07  (0.93,1.24) | **0.76***  **(0.64,0.91)** |
| Lag06 | 1.18  (0.99,1.42) | 1.14  (0.95,1.38) | **1.20***  **(1.06,1.37)** | **1.32***  **(1.09,1.61)** | 1.08  (0.93,1.26) | **0.74***  **(0.62,0.89)** |
| Female | | | | | | |
| Lag01 | 0.99  (0.83,1.18) | 0.97  (0.81,1.17) | 1.01  (0.86,1.19) | 1.03  (0.85,1.24) | 0.97  (0.83,1.13) | **0.78***  **(0.63,0.98)** |
| Lag02 | 1.00  (0.82,1.21) | 0.97  (0.79,1.20) | 1.05  (0.89,1.25) | 1.02  (0.82,1.26) | 0.96  (0.81,1.14) | **0.73***  **(0.58,0.93)** |
| Lag03 | 1.05  (0.85,1.29) | 1.01  (0.81,1.27) | 1.09  (0.91,1.31) | 1.06  (0.84,1.34) | 0.98  (0.81,1.19) | **0.71***  **(0.56,0.92)** |
| Lag04 | 1.07  (0.85,1.35) | 1.03  (0.81,1.32) | 1.13  (0.94,1.36) | 1.12  (0.87,1.44) | 0.99  (0.81,1.22) | **0.71***  **(0.55,0.92)** |
| Lag05 | 1.07  (0.83,1.38) | 1.05  (0.81,1.36) | 1.11  (0.92,1.35) | 1.14  (0.87,1.49) | 0.98  (0.78,1.22) | **0.72***  **(0.56,0.93)** |
| Lag06 | 1.07  (0.82,1.41) | 1.05  (0.79,1.39) | 1.10  (0.90,1.34) | 1.12  (0.84,1.50) | 0.95  (0.75,1.21) | **0.75***  **(0.58,0.97)** |

Notes: * and bold indicate p value<0.05. The interquartile range (IQR) of daily concentrations of PM_2.5_, PM_10_, SO_2_, NO_2_, CO, and O_3_ were 77.50μg/m^3^, 88.94μg/m^3^, 17.40μg/m^3^, 28.36μg/m^3^, 0.88mg/m^3^ and 64.15μg/m^3^, respectively.

**Supplementary Table 2.** The relative risk (RR) of hospital admission for asthma in children per interquartile range (IQR) increase in different air pollutants in different moving-average lags: single-pollutant model, stratified by age

|  | **PM_2.5_** | **PM_10_** | **SO_2_** | **NO_2_** | **CO** | **O_3_** |
| --- | --- | --- | --- | --- | --- | --- |
|  | **RR (95%CI)** | **RR (95%CI)** | **RR (95%CI)** | **RR (95%CI)** | **RR (95%CI)** | **RR(95%CI)** |
| Preschool children | | | | | | |
| Lag01 | 0.99  (0.88,1.12) | 0.99  (0.87,1.12) | 1.07  (0.97,1.18) | 1.04  (0.91,1.18) | 0.95  (0.85,1.05) | **0.76***  **(0.63,0.90)** |
| Lag02 | 1.05  (0.93,1.20) | 1.05  (0.91,1.20) | **1.13***  **(1.01,1.25)** | 1.10  (0.95,1.27) | 1.00  (0.89,1.12) | **0.74***  **(0.62,0.89)** |
| Lag03 | 1.09  (0.95,1.26) | 1.10  (0.95,1.28) | **1.17***  **(1.05,1.31)** | 1.17  (1.00,1.36) | 1.02  (0.9,1.16) | **0.73***  **(0.61,0.89)** |
| Lag04 | 1.14  (0.98,1.33) | 1.15  (0.98,1.35) | **1.23***  **(1.09,1.38)** | **1.24***  **(1.05,1.47)** | 1.05  (0.92,1.2) | **0.70***  **(0.57,0.85)** |
| Lag05 | 1.16  (0.98,1.38) | 1.18  (0.99,1.40) | **1.23***  **(1.09,1.39)** | **1.28***  **(1.07,1.53)** | 1.04  (0.9,1.21) | **0.68***  **(0.55,0.83)** |
| Lag06 | 1.19  (0.99,1.43) | 1.20  (0.99,1.45) | **1.23***  **(1.08,1.39)** | **1.29***  **(1.06,1.56)** | 1.04  (0.89,1.21) | **0.67***  **(0.54,0.82)** |
| School-aged children | | | | | | |
| Lag01 | 0.92  (0.76,1.10) | 0.84  (0.69,1.02) | 0.92  (0.76,1.11) | 0.97  (0.80,1.19) | 0.93  (0.79,1.10) | 0.85  (0.70,1.04) |
| Lag02 | 0.96  (0.78,1.17) | 0.87  (0.70,1.08) | 0.93  (0.76,1.14) | 0.98  (0.78,1.23) | 0.96  (0.80,1.15) | 0.85  (0.69,1.05) |
| Lag03 | 0.99  (0.79,1.23) | 0.90  (0.71,1.14) | 0.97  (0.78,1.19) | 1.03  (0.81,1.32) | 0.98  (0.80,1.19) | 0.85  (0.69,1.05) |
| Lag04 | 1.01  (0.79,1.28) | 0.90  (0.70,1.16) | 0.96  (0.77,1.20) | 1.06  (0.81,1.38) | 1.00  (0.80,1.24) | 0.86  (0.69,1.06) |
| Lag05 | 1.04  (0.80,1.34) | 0.92  (0.70,1.21) | 0.97  (0.78,1.22) | 1.12  (0.84,1.49) | 1.03  (0.82,1.29) | 0.86  (0.69,1.07) |
| Lag06 | 1.04  (0.79,1.38) | 0.92  (0.69,1.23) | 0.97  (0.77,1.23) | 1.17  (0.86,1.58) | 1.04  (0.81,1.33) | 0.87  (0.70,1.08) |

Notes: * and bold indicate p value<0.05. The interquartile range (IQR) of daily concentrations of PM_2.5_, PM_10_, SO_2_, NO_2_, CO, and O_3_ were 77.50μg/m^3^, 88.94μg/m^3^, 17.40μg/m^3^, 28.36μg/m^3^, 0.88mg/m^3^ and 64.15μg/m^3^, respectively.

**Supplementary Table 3.** The relative risk (RR) of hospital admission for asthma in children per interquartile range (IQR) increase in different air pollutants in different moving-average lags: single-pollutant model, stratified by season

|  | **PM_2.5_** | **PM_10_** | **SO_2_** | **NO_2_** | **CO** | **O_3_** |
| --- | --- | --- | --- | --- | --- | --- |
|  | **RR (95%CI)** | **RR (95%CI)** | **RR (95%CI)** | **RR (95%CI)** | **RR (95%CI)** | **RR(95%CI)** |
| Cold season | | | | | | |
| Lag01 | 0.92  (0.80,1.06) | 0.90  (0.77,1.05) | 1.09  (0.98,1.20) | 0.93  (0.81,1.08) | 0.90  (0.81,1.01) | 0.67  (0.44,1.01) |
| Lag02 | 1.00  (0.86,1.16) | 0.97  (0.82,1.14) | **1.16***  **(1.04,1.29)** | 1.00  (0.85,1.18) | 0.96  (0.85,1.08) | 0.65  (0.42,1.00) |
| Lag03 | 1.05  (0.89,1.24) | 1.03  (0.86,1.23) | **1.22***  **(1.10,1.36)** | 1.07  (0.90,1.29) | 0.99  (0.87,1.13) | **0.60***  **(0.38,0.94)** |
| Lag04 | 1.09  (0.91,1.30) | 1.07  (0.88,1.29) | **1.28***  **(1.14,1.43)** | 1.13  (0.93,1.37) | 1.02  (0.88,1.17) | **0.56***  **(0.35,0.90)** |
| Lag05 | 1.09  (0.90,1.33) | 1.09  (0.89,1.34) | **1.30***  **(1.16,1.47)** | 1.16  (0.94,1.43) | 1.01  (0.87,1.18) | **0.54***  **(0.33,0.87)** |
| Lag06 | 1.09  (0.88,1.35) | 1.10  (0.88,1.38) | **1.32***  **(1.16,1.49)** | 1.16  (0.92,1.45) | 1.01  (0.86,1.19) | **0.55***  **(0.34,0.90)** |
| Warm season | | | | | | |
| Lag01 | 1.05  (0.90,1.22) | 1.02  (0.86,1.20) | 0.94  (0.76,1.18) | **1.21***  **(1.01,1.44)** | 1.07  (0.91,1.27) | **0.79***  **(0.69,0.92)** |
| Lag02 | 1.10  (0.93,1.30) | 1.05  (0.88,1.27) | 0.98  (0.77,1.24) | 1.22  (1.00,1.49) | 1.12  (0.93,1.34) | **0.79***  **(0.68,0.92)** |
| Lag03 | 1.13  (0.94,1.37) | 1.10  (0.91,1.35) | 1.00  (0.78,1.29) | **1.27***  **(1.02,1.58)** | 1.13  (0.93,1.39) | **0.80***  **(0.69,0.93)** |
| Lag04 | 1.20  (0.97,1.47) | 1.15  (0.93,1.42) | 1.07  (0.82,1.38) | **1.35***  **(1.07,1.70)** | 1.19  (0.96,1.48) | **0.79***  **(0.67,0.93)** |
| Lag05 | **1.27***  **(1.01,1.59)** | 1.21  (0.96,1.52) | 1.06  (0.81,1.39) | **1.43***  **(1.12,1.83)** | 1.24  (0.99,1.57) | **0.78***  **(0.67,0.92)** |
| Lag06 | **1.34***  **(1.05,1.71)** | 1.25  (0.98,1.59) | 1.06  (0.81,1.40) | **1.47***  **(1.13,1.91)** | 1.26  (0.98,1.62) | **0.78***  **(0.66,0.92)** |

Notes: * and bold indicate p value<0.05. The interquartile range (IQR) of daily concentrations of PM_2.5_, PM_10_, SO_2_, NO_2_, CO, and O_3_ were 77.50μg/m^3^, 88.94μg/m^3^, 17.40μg/m^3^, 28.36μg/m^3^, 0.88mg/m^3^ and 64.15μg/m^3^, respectively.

**Supplementary Table 4.** The relative risk (RR) of hospital admission for asthma in children per IQR increase in different pollutants with adjustment for different lag days of temperature

|  |  | **Lag day of temperature** | | | | | |
| --- | --- | --- | --- | --- | --- | --- | --- |
|  |  | **Lag1** | **Lag2** | **Lag3** | **Lag4** | **Lag5** | **Lag6** |
|  |  | **RR (95%CI)** | **RR (95%CI)** | **RR (95%CI)** | **RR (95%CI)** | **RR (95%CI)** | **RR (95%CI)** |
| PM_2.5_ | Lag0 | 0.93  (0.85,1.02) | 0.93  (0.85,1.02) | 0.93  (0.85,1.02) | 0.93  (0.85,1.03) | 0.94  (0.85,1.03) | 0.93  (0.85,1.03) |
|  | Lag1 | 1.02  (0.94,1.11) | 1.03  (0.94,1.12) | 1.02  (0.94,1.12) | 1.03  (0.94,1.12) | 1.03  (0.94,1.13) | 1.03  (0.94,1.13) |
|  | Lag2 | **1.09***  **(1.00,1.19)** | **1.10***  **(1.01,1.19)** | **1.10***  **(1.01,1.20)** | **1.10***  **(1.01,1.20)** | **1.10***  **(1.01,1.20)** | **1.10***  **(1.01,1.20)** |
|  | Lag3 | 1.07  (0.99,1.17) | 1.07  (0.98,1.17) | 1.08  (0.99,1.17) | 1.08  (0.99,1.18) | 1.08  (0.99,1.18) | 1.08  (0.99,1.18) |
|  | Lag4 | 1.07  (0.99,1.17) | 1.07  (0.99,1.17) | 1.07  (0.98,1.16) | 1.07  (0.99,1.17) | 1.08  (0.99,1.18) | 1.08  (0.99,1.17) |
|  | Lag5 | 1.05  (0.97,1.15) | 1.06  (0.97,1.15) | 1.05  (0.97,1.15) | 1.04  (0.96,1.14) | 1.05  (0.97,1.15) | 1.06  (0.97,1.15) |
|  | Lag6 | 1.04  (0.96,1.14) | 1.05  (0.96,1.15) | 1.05  (0.96,1.14) | 1.04  (0.96,1.14) | 1.04  (0.95,1.13) | 1.05  (0.96,1.14) |
|  | Lag01 | 0.97  (0.88,1.08) | 0.98  (0.88,1.08) | 0.97  (0.88,1.08) | 0.98  (0.88,1.08) | 0.98  (0.88,1.09) | 0.98  (0.88,1.09) |
|  | Lag02 | 1.03  (0.92,1.15) | 1.03  (0.92,1.16) | 1.03  (0.92,1.16) | 1.04  (0.92,1.16) | 1.04  (0.93,1.17) | 1.04  (0.93,1.17) |
|  | Lag03 | 1.06  (0.94,1.20) | 1.07  (0.94,1.21) | 1.07  (0.95,1.21) | 1.08  (0.95,1.22) | 1.08  (0.95,1.23) | 1.08  (0.95,1.23) |
|  | Lag04 | 1.10  (0.96,1.26) | 1.11  (0.97,1.26) | 1.11  (0.97,1.27) | 1.12  (0.97,1.28) | 1.12  (0.98,1.29) | 1.12  (0.97,1.29) |
|  | Lag05 | 1.12  (0.97,1.30) | 1.13  (0.98,1.31) | 1.13  (0.98,1.31) | 1.13  (0.98,1.31) | 1.15  (0.99,1.33) | 1.15  (0.99,1.33) |
|  | Lag06 | 1.14  (0.98,1.33) | 1.15  (0.99,1.35) | 1.15  (0.99,1.35) | 1.15  (0.99,1.35) | 1.16  (0.99,1.36) | 1.17  (0.99,1.37) |
| PM_10_ | Lag0 | 0.91  (0.83,1.00) | 0.91  (0.82,1.00) | **0.90***  **(0.82,1.00)** | **0.90***  **(0.82,1.00)** | 0.91  (0.82,1.00) | **0.90***  **(0.82,1.00)** |
|  | Lag1 | 1.00  (0.92,1.10) | 1.01  (0.92,1.11) | 1.00  (0.91,1.10) | 1.00  (0.91,1.10) | 1.00  (0.91,1.10) | 1.00  (0.91,1.10) |
|  | Lag2 | 1.08  (0.99,1.18) | 1.09  (0.99,1.19) | 1.09  (0.99,1.19) | 1.08  (0.99,1.18) | 1.08  (0.99,1.19) | 1.08  (0.99,1.19) |
|  | Lag3 | 1.09  (1.00,1.19) | 1.09  (1.00,1.19) | **1.09***  **(1.00,1.20)** | 1.09  (1.00,1.20) | 1.09  (1.00,1.19) | 1.09  (1.00,1.19) |
|  | Lag4 | 1.06  (0.97,1.16) | 1.06  (0.97,1.16) | 1.06  (0.97,1.16) | 1.06  (0.97,1.16) | 1.06  (0.97,1.17) | 1.06  (0.97,1.16) |
|  | Lag5 | 1.04  (0.95,1.14) | 1.04  (0.95,1.14) | 1.04  (0.95,1.14) | 1.02  (0.93,1.12) | 1.04  (0.95,1.14) | 1.04  (0.95,1.14) |
|  | Lag6 | 1.03  (0.94,1.13) | 1.03  (0.94,1.13) | 1.03  (0.94,1.13) | 1.02  (0.93,1.12) | 1.02  (0.93,1.12) | 1.03  (0.94,1.13) |
|  | Lag01 | 0.95  (0.85,1.06) | 0.95  (0.85,1.06) | 0.94  (0.85,1.05) | 0.94  (0.84,1.05) | 0.94  (0.84,1.05) | 0.94  (0.84,1.05) |
|  | Lag02 | 1.00  (0.88,1.12) | 1.00  (0.89,1.13) | 1.00  (0.88,1.13) | 0.99  (0.87,1.12) | 0.99  (0.88,1.12) | 0.99  (0.88,1.12) |
|  | Lag03 | 1.04  (0.92,1.19) | 1.05  (0.92,1.19) | 1.05  (0.92,1.19) | 1.04  (0.91,1.19) | 1.04  (0.91,1.19) | 1.04  (0.91,1.19) |
|  | Lag04 | 1.07  (0.93,1.23) | 1.07  (0.93,1.24) | 1.07  (0.93,1.24) | 1.07  (0.92,1.23) | 1.07  (0.93,1.24) | 1.07  (0.92,1.24) |
|  | Lag05 | 1.09  (0.94,1.27) | 1.10  (0.95,1.28) | 1.10  (0.94,1.28) | 1.09  (0.93,1.27) | 1.10  (0.94,1.28) | 1.10  (0.94,1.28) |
|  | Lag06 | 1.11  (0.94,1.30) | 1.12  (0.95,1.31) | 1.11  (0.95,1.31) | 1.10  (0.93,1.30) | 1.11  (0.94,1.31) | 1.11  (0.94,1.31) |
| SO_2_ | Lag0 | 1.01  (0.92,1.10) | 1.01  (0.92,1.10) | 1.01  (0.93,1.10) | 1.02  (0.93,1.12) | 1.02  (0.94,1.12) | 1.02  (0.93,1.11) |
|  | Lag1 | 1.06  (0.97,1.15) | 1.07  (0.98,1.16) | 1.07  (0.98,1.16) | 1.08  (0.99,1.17) | 1.08  (0.99,1.18) | 1.08  (0.99,1.18) |
|  | Lag2 | **1.11***  **(1.02,1.20)** | **1.12***  **(1.04,1.21)** | **1.13***  **(1.05,1.23)** | **1.14***  **(1.05,1.23)** | **1.14***  **(1.05,1.24)** | **1.14***  **(1.05,1.24)** |
|  | Lag3 | **1.12***  **(1.04,1.21)** | **1.13***  **(1.04,1.22)** | **1.14***  **(1.05,1.23)** | **1.16***  **(1.07,1.25)** | **1.15***  **(1.07,1.25)** | **1.15***  **(1.06,1.25)** |
|  | Lag4 | **1.14***  **(1.05,1.22)** | **1.14***  **(1.05,1.22)** | **1.14***  **(1.05,1.22)** | **1.15***  **(1.07,1.24)** | **1.16***  **(1.08,1.26)** | **1.16***  **(1.07,1.25)** |
|  | Lag5 | 1.06  (0.98,1.15) | 1.07  (0.98,1.15) | 1.07  (0.98,1.15) | 1.06  (0.98,1.15) | 1.08  (0.99,1.17) | 1.08  (1.00,1.18) |
|  | Lag6 | 1.05  (0.97,1.14) | 1.06  (0.97,1.15) | 1.06  (0.98,1.15) | 1.06  (0.97,1.14) | 1.05  (0.97,1.14) | 1.06  (0.98,1.15) |
|  | Lag01 | 1.04  (0.95,1.14) | 1.05  (0.95,1.15) | 1.05  (0.96,1.16) | 1.07  (0.97,1.17) | 1.07  (0.97,1.18) | 1.06  (0.96,1.17) |
|  | Lag02 | 1.08  (0.98,1.20) | 1.10  (0.99,1.21) | **1.11***  **(1.00,1.23)** | **1.12***  **(1.02,1.25)** | **1.13***  **(1.02,1.25)** | **1.12***  **(1.01,1.25)** |
|  | Lag03 | **1.12***  **(1.02,1.24)** | **1.14***  **(1.03,1.26)** | **1.15***  **(1.04,1.28)** | **1.18***  **(1.06,1.31)** | **1.18***  **(1.06,1.31)** | **1.18***  **(1.05,1.31)** |
|  | Lag04 | **1.16***  **(1.05,1.29)** | **1.18***  **(1.06,1.31)** | **1.19***  **(1.07,1.32)** | **1.22***  **(1.09,1.36)** | **1.23***  **(1.1,1.38)** | **1.23***  **(1.10,1.37)** |
|  | Lag05 | **1.17***  **(1.05,1.30)** | **1.18***  **(1.06,1.32)** | **1.19***  **(1.07,1.33)** | **1.21***  **(1.09,1.36)** | **1.23***  **(1.1,1.38)** | **1.23***  **(1.10,1.39)** |
|  | Lag06 | **1.17***  **(1.04,1.31)** | **1.18***  **(1.05,1.32)** | **1.19***  **(1.06,1.33)** | **1.21***  **(1.07,1.35)** | **1.22***  **(1.08,1.37)** | **1.23***  **(1.09,1.38)** |
| NO_2_ | Lag0 | 1.00  (0.90,1.10) | 1.00  (0.90,1.10) | 1.01  (0.91,1.11) | 1.01  (0.91,1.11) | 1.01  (0.92,1.12) | 1.01  (0.91,1.11) |
|  | Lag1 | 1.04  (0.95,1.15) | 1.05  (0.96,1.16) | 1.06  (0.96,1.17) | 1.06  (0.96,1.17) | 1.07  (0.96,1.18) | 1.06  (0.96,1.17) |
|  | Lag2 | 1.08  (0.98,1.19) | 1.09  (0.99,1.20) | **1.10***  **(1.00,1.22)** | 1.10  (1.00,1.21) | **1.11***  **(1.00,1.22)** | **1.11***  **(1.00,1.22)** |
|  | Lag3 | **1.12***  **(1.02,1.23)** | **1.12***  **(1.02,1.24)** | **1.13***  **(1.03,1.25)** | **1.14***  **(1.04,1.26)** | **1.14***  **(1.04,1.26)** | **1.14***  **(1.04,1.26)** |
|  | Lag4 | **1.12***  **(1.02,1.24)** | **1.12***  **(1.02,1.24)** | **1.12***  **(1.02,1.23)** | **1.12***  **(1.02,1.23)** | **1.14***  **(1.03,1.25)** | **1.13***  **(1.03,1.25)** |
|  | Lag5 | **1.11***  **(1.01,1.22)** | **1.11***  **(1.01,1.22)** | **1.11***  **(1.01,1.22)** | **1.09**  **(0.99,1.20)** | **1.11***  **(1.01,1.22)** | **1.12***  **(1.01,1.23)** |
|  | Lag6 | 1.06  (0.96,1.17) | 1.07  (0.97,1.18) | 1.07  (0.97,1.18) | 1.06  (0.96,1.16) | 1.05  (0.95,1.16) | 1.06  (0.96,1.17) |
|  | Lag01 | 1.03  (0.92,1.15) | 1.04  (0.93,1.16) | 1.04  (0.93,1.17) | 1.05  (0.93,1.17) | 1.05  (0.94,1.18) | 1.05  (0.93,1.17) |
|  | Lag02 | 1.07  (0.94,1.21) | 1.08  (0.96,1.23) | 1.10  (0.97,1.24) | 1.10  (0.97,1.25) | 1.10  (0.97,1.25) | 1.10  (0.97,1.25) |
|  | Lag03 | 1.12  (0.98,1.29) | 1.14  (1.00,1.31) | **1.16***  **(1.01,1.33)** | **1.17***  **(1.01,1.34)** | **1.17***  **(1.02,1.35)** | **1.17***  **(1.02,1.34)** |
|  | Lag04 | **1.18***  **(1.02,1.37)** | **1.20***  **(1.03,1.39)** | **1.21***  **(1.05,1.41)** | **1.22***  **(1.05,1.42)** | **1.24***  **(1.07,1.44)** | **1.23***  **(1.06,1.43)** |
|  | Lag05 | **1.22***  **(1.05,1.43)** | **1.24***  **(1.06,1.45)** | **1.26***  **(1.08,1.47)** | **1.25***  **(1.07,1.47)** | **1.28***  **(1.09,1.5)** | **1.28***  **(1.09,1.50)** |
|  | Lag06 | **1.24***  **(1.05,1.47)** | **1.26***  **(1.07,1.49)** | **1.28***  **(1.08,1.51)** | **1.26***  **(1.07,1.49)** | **1.29***  **(1.09,1.52)** | **1.29***  **(1.09,1.53)** |
| CO | Lag0 | **0.92***  **(0.84,1.00)** | 0.92  (0.85,1.00) | 0.92  (0.85,1.01) | 0.94  (0.86,1.02) | 0.94  (0.86,1.02) | 0.93  (0.85,1.02) |
|  | Lag1 | 0.99  (0.91,1.07) | 1.00  (0.92,1.08) | 1.00  (0.92,1.09) | 1.01  (0.94,1.10) | 1.02  (0.94,1.10) | 1.01  (0.93,1.10) |
|  | Lag2 | 1.06  (0.98,1.14) | 1.07  (0.99,1.15) | **1.08***  **(1.00,1.16)** | **1.09***  **(1.01,1.17)** | **1.09***  **(1.01,1.18)** | **1.10***  **(1.02,1.18)** |
|  | Lag3 | 1.04  (0.96,1.12) | 1.04  (0.96,1.12) | 1.04  (0.97,1.12) | 1.06  (0.98,1.14) | 1.06  (0.98,1.14) | 1.06  (0.98,1.14) |
|  | Lag4 | 1.04  (0.97,1.12) | 1.04  (0.97,1.12) | 1.04  (0.96,1.12) | 1.05  (0.98,1.13) | 1.06  (0.98,1.14) | 1.06  (0.98,1.14) |
|  | Lag5 | 1.01  (0.94,1.10) | 1.02  (0.94,1.10) | 1.01  (0.94,1.10) | 1.01  (0.94,1.09) | 1.02  (0.94,1.10) | 1.02  (0.95,1.11) |
|  | Lag6 | 1.00  (0.92,1.08) | 1.01  (0.93,1.09) | 1.01  (0.93,1.09) | 1.01  (0.93,1.09) | 1.00  (0.93,1.08) | 1.01  (0.93,1.09) |
|  | Lag01 | 0.94  (0.86,1.04) | 0.95  (0.87,1.04) | 0.96  (0.87,1.05) | 0.97  (0.88,1.07) | 0.97  (0.88,1.07) | 0.97  (0.88,1.06) |
|  | Lag02 | 0.99  (0.90,1.09) | 1.00  (0.90,1.10) | 1.01  (0.91,1.12) | 1.03  (0.93,1.14) | 1.03  (0.93,1.15) | 1.03  (0.92,1.14) |
|  | Lag03 | 1.01  (0.91,1.12) | 1.02  (0.91,1.13) | 1.03  (0.92,1.15) | 1.06  (0.95,1.18) | 1.06  (0.95,1.19) | 1.06  (0.94,1.19) |
|  | Lag04 | 1.03  (0.92,1.15) | 1.04  (0.93,1.17) | 1.05  (0.93,1.18) | 1.08  (0.96,1.22) | 1.09  (0.96,1.23) | 1.08  (0.96,1.23) |
|  | Lag05 | 1.03  (0.91,1.17) | 1.05  (0.92,1.18) | 1.05  (0.93,1.19) | 1.08  (0.95,1.23) | 1.09  (0.96,1.25) | 1.09  (0.96,1.25) |
|  | Lag06 | 1.03  (0.90,1.18) | 1.05  (0.91,1.19) | 1.05  (0.92,1.21) | 1.08  (0.94,1.24) | 1.09  (0.95,1.25) | 1.09  (0.95,1.26) |
| O_3_ | Lag0 | **0.84***  **(0.75,0.95)** | **0.83***  **(0.74,0.93)** | **0.82***  **(0.73,0.92)** | **0.82***  **(0.73,0.92)** | **0.82***  **(0.73,0.92)** | **0.83***  **(0.74,0.93)** |
|  | Lag1 | **0.81***  **(0.71,0.92)** | **0.80***  **(0.71,0.90)** | **0.79***  **(0.70,0.89)** | **0.79***  **(0.70,0.89)** | **0.79***  **(0.70,0.89)** | **0.80***  **(0.71,0.89)** |
|  | Lag2 | **0.86***  **(0.76,0.97)** | **0.84***  **(0.75,0.96)** | **0.83***  **(0.74,0.94)** | **0.83***  **(0.74,0.93)** | **0.83***  **(0.74,0.93)** | **0.84***  **(0.75,0.94)** |
|  | Lag3 | **0.87***  **(0.78,0.98)** | **0.86***  **(0.77,0.97)** | **0.85***  **(0.75,0.96)** | **0.85***  **(0.76,0.96)** | **0.85***  **(0.75,0.95)** | **0.85***  **(0.76,0.95)** |
|  | Lag4 | **0.82***  **(0.73,0.92)** | **0.81***  **(0.72,0.91)** | **0.80***  **(0.71,0.91)** | **0.80***  **(0.71,0.91)** | **0.80***  **(0.71,0.90)** | **0.80***  **(0.72,0.91)** |
|  | Lag5 | **0.83***  **(0.74,0.93)** | **0.82***  **(0.73,0.92)** | **0.81***  **(0.72,0.92)** | **0.82***  **(0.73,0.93)** | **0.81***  **(0.72,0.92)** | **0.82***  **(0.72,0.92)** |
|  | Lag6 | **0.85***  **(0.75,0.95)** | **0.84***  **(0.74,0.94)** | **0.83***  **(0.74,0.93)** | **0.83***  **(0.74,0.94)** | **0.83***  **(0.74,0.94)** | **0.84***  **(0.74,0.95)** |
|  | Lag01 | **0.80***  **(0.70,0.92)** | **0.79***  **(0.69,0.90)** | **0.78***  **(0.69,0.89)** | **0.78***  **(0.69,0.89)** | **0.78***  **(0.69,0.88)** | **0.79***  **(0.70,0.89)** |
|  | Lag02 | **0.79***  **(0.69,0.91)** | **0.78***  **(0.68,0.89)** | **0.77***  **(0.67,0.88)** | **0.77***  **(0.68,0.88)** | **0.77***  **(0.68,0.88)** | **0.78***  **(0.69,0.89)** |
|  | Lag03 | **0.79***  **(0.68,0.91)** | **0.77***  **(0.67,0.90)** | **0.76***  **(0.66,0.88)** | **0.77***  **(0.67,0.88)** | **0.77***  **(0.67,0.88)** | **0.78***  **(0.68,0.89)** |
|  | Lag04 | **0.77***  **(0.67,0.90)** | **0.76***  **(0.65,0.88)** | **0.74***  **(0.64,0.86)** | **0.75***  **(0.65,0.87)** | **0.75***  **(0.65,0.86)** | **0.76***  **(0.66,0.87)** |
|  | Lag05 | **0.76***  **(0.65,0.89)** | **0.74***  **(0.64,0.87)** | **0.73***  **(0.63,0.85)** | **0.74***  **(0.64,0.86)** | **0.73***  **(0.63,0.85)** | **0.75***  **(0.65,0.86)** |
|  | Lag06 | **0.76***  **(0.65,0.88)** | **0.74***  **(0.63,0.86)** | **0.73***  **(0.62,0.85)** | **0.73***  **(0.63,0.85)** | **0.73***  **(0.63,0.85)** | **0.74***  **(0.64,0.86)** |

Notes: * and bold indicate *p* value <0.05. Tmean, daily mean temperature. The interquartile range (IQR) of daily concentrations of PM_2.5_, PM_10_, SO_2_, NO_2_, CO, and O_3_ were 77.50μg/m^3^, 88.94μg/m^3^, 17.40μg/m^3^, 28.36μg/m^3^, 0.88mg/m^3^ and 64.15μg/m^3^, respectively.

**Supplementary Table 5.** The relative risk (RR) of hospital admission for asthma in children per IQR increase in different pollutants with adjustment for different dfs of nature cubic spline of daily mean temperature

|  |  | **df=3** | **df=4** | **df=5** | **df=6** |
| --- | --- | --- | --- | --- | --- |
|  |  | **RR (95% CI)** | **RR (95% CI)** | **RR (95% CI)** | **RR (95% CI)** |
| PM_2.5_ | Lag0 | 0.93(0.85,1.02) | 0.93(0.85,1.02) | 0.93(0.85,1.03) | 0.94(0.85,1.03) |
|  | Lag1 | 1.02(0.93,1.11) | 1.02(0.93,1.11) | 1.02(0.93,1.11) | 1.02(0.93,1.11) |
|  | Lag2 | **1.10*(1.01,1.19)** | **1.09*(1.01,1.19)** | **1.10*(1.01,1.19)** | **1.10*(1.01,1.19)** |
|  | Lag3 | 1.08(0.99,1.17) | 1.08(0.99,1.17) | 1.08(0.99,1.17) | 1.08(0.99,1.18) |
|  | Lag4 | 1.08(0.99,1.17) | 1.07(0.99,1.17) | 1.08(0.99,1.18) | 1.08(0.99,1.18) |
|  | Lag5 | 1.05(0.97,1.15) | 1.05(0.97,1.15) | 1.06(0.97,1.15) | 1.06(0.97,1.16) |
|  | Lag6 | 1.05(0.96,1.14) | 1.04(0.96,1.14) | 1.05(0.96,1.15) | 1.05(0.97,1.15) |
|  | Lag01 | 0.97(0.87,1.07) | 0.97(0.88,1.08) | 0.97(0.88,1.08) | 0.97(0.88,1.08) |
|  | Lag02 | 1.03(0.92,1.15) | 1.03(0.92,1.15) | 1.03(0.92,1.15) | 1.03(0.92,1.15) |
|  | Lag03 | 1.06(0.94,1.20) | 1.06(0.94,1.20) | 1.07(0.94,1.21) | 1.07(0.94,1.21) |
|  | Lag04 | 1.10(0.96,1.26) | 1.10(0.96,1.26) | 1.11(0.97,1.27) | 1.11(0.97,1.27) |
|  | Lag05 | 1.13(0.97,1.30) | 1.12(0.97,1.30) | 1.13(0.98,1.31) | 1.14(0.98,1.32) |
|  | Lag06 | 1.15(0.98,1.34) | 1.15(0.98,1.34) | 1.16(0.99,1.36) | 1.17(0.99,1.37) |
| PM_10_ | Lag0 | 0.91(0.83,1.00) | 0.91(0.82,1.00) | 0.91(0.83,1.00) | 0.91(0.83,1.01) |
|  | Lag1 | 1.00(0.91,1.10) | 1.00(0.91,1.10) | 1.00(0.91,1.10) | 1.00(0.91,1.10) |
|  | Lag2 | 1.08(0.99,1.18) | 1.08(0.99,1.18) | 1.08(0.99,1.18) | 1.08(0.99,1.18) |
|  | Lag3 | 1.09(1.00,1.19) | 1.09(1.00,1.19) | 1.09(1.00,1.20) | 1.10*(1.00,1.20) |
|  | Lag4 | 1.06(0.97,1.16) | 1.06(0.97,1.16) | 1.07(0.97,1.17) | 1.07(0.98,1.17) |
|  | Lag5 | 1.04(0.95,1.14) | 1.04(0.95,1.14) | 1.04(0.95,1.14) | 1.05(0.95,1.15) |
|  | Lag6 | 1.03(0.94,1.13) | 1.03(0.94,1.13) | 1.03(0.94,1.13) | 1.04(0.94,1.14) |
|  | Lag01 | 0.94(0.84,1.05) | 0.94(0.84,1.05) | 0.94(0.85,1.05) | 0.94(0.84,1.06) |
|  | Lag02 | 0.99(0.88,1.12) | 0.99(0.88,1.12) | 0.99(0.88,1.12) | 1.00(0.88,1.12) |
|  | Lag03 | 1.04(0.91,1.19) | 1.04(0.91,1.19) | 1.04(0.91,1.19) | 1.05(0.92,1.19) |
|  | Lag04 | 1.07(0.93,1.24) | 1.07(0.93,1.23) | 1.08(0.93,1.24) | 1.08(0.93,1.25) |
|  | Lag05 | 1.10(0.94,1.28) | 1.09(0.94,1.27) | 1.10(0.94,1.28) | 1.11(0.95,1.29) |
|  | Lag06 | 1.11(0.94,1.31) | 1.11(0.94,1.30) | 1.12(0.95,1.32) | 1.12(0.95,1.33) |
| SO_2_ | Lag0 | 1.00(0.92,1.09) | 1.01(0.93,1.10) | 1.01(0.93,1.10) | 1.01(0.93,1.10) |
|  | Lag1 | 1.05(0.97,1.14) | 1.06(0.97,1.15) | 1.06(0.98,1.15) | 1.06(0.97,1.15) |
|  | Lag2 | **1.11*(1.03,1.20)** | **1.12*(1.04,1.21)** | **1.12*(1.04,1.21)** | **1.12*(1.03,1.21)** |
|  | Lag3 | **1.13*(1.05,1.22)** | **1.14*(1.05,1.23)** | **1.14*(1.05,1.23)** | **1.13*(1.05,1.23)** |
|  | Lag4 | **1.14*(1.06,1.23)** | **1.15*(1.06,1.24)** | **1.15*(1.07,1.24)** | **1.15*(1.06,1.24)** |
|  | Lag5 | 1.07(0.98,1.16) | 1.07(0.99,1.16) | 1.08(0.99,1.17) | 1.08(0.99,1.17) |
|  | Lag6 | 1.05(0.97,1.14) | 1.06(0.98,1.15) | 1.06(0.98,1.15) | 1.06(0.98,1.15) |
|  | Lag01 | 1.03(0.94,1.14) | 1.04(0.95,1.14) | 1.04(0.95,1.15) | 1.04(0.95,1.14) |
|  | Lag02 | 1.08(0.98,1.19) | 1.09(0.99,1.21) | 1.09(0.99,1.21) | 1.09(0.99,1.20) |
|  | Lag03 | **1.12*(1.02,1.24)** | **1.14*(1.03,1.26)** | **1.14*(1.03,1.26)** | **1.13*(1.02,1.26)** |
|  | Lag04 | **1.16*(1.05,1.29)** | **1.18*(1.06,1.32)** | **1.18*(1.06,1.32)** | **1.18*(1.06,1.31)** |
|  | Lag05 | **1.17*(1.05,1.31)** | **1.19*(1.06,1.33)** | **1.19*(1.07,1.33)** | **1.19*(1.06,1.33)** |
|  | Lag06 | **1.17*(1.04,1.31)** | **1.19*(1.06,1.33)** | **1.19*(1.06,1.34)** | **1.19*(1.06,1.33)** |
| NO_2_ | Lag0 | 0.99(0.90,1.09) | 0.99(0.90,1.09) | 0.99(0.90,1.10) | 1.00(0.91,1.10) |
|  | Lag1 | 1.04(0.94,1.14) | 1.04(0.94,1.14) | 1.04(0.94,1.15) | 1.04(0.94,1.15) |
|  | Lag2 | 1.09(0.99,1.20) | 1.08(0.99,1.19) | 1.09(0.99,1.20) | 1.08(0.98,1.19) |
|  | Lag3 | **1.13*(1.03,1.25)** | **1.13*(1.03,1.24)** | **1.13*(1.03,1.24)** | **1.13*(1.03,1.25)** |
|  | Lag4 | **1.13*(1.03,1.24)** | **1.13*(1.03,1.24)** | **1.13*(1.03,1.25)** | **1.13*(1.03,1.25)** |
|  | Lag5 | **1.11*(1.01,1.23)** | **1.11*(1.01,1.23)** | **1.12*(1.01,1.23)** | **1.12*(1.02,1.23)** |
|  | Lag6 | 1.06(0.96,1.17) | 1.06(0.96,1.17) | 1.06(0.96,1.17) | 1.07(0.97,1.18) |
|  | Lag01 | 1.02(0.91,1.14) | 1.02(0.91,1.14) | 1.02(0.92,1.15) | 1.03(0.92,1.15) |
|  | Lag02 | 1.07(0.94,1.21) | 1.07(0.94,1.21) | 1.07(0.94,1.21) | 1.07(0.94,1.21) |
|  | Lag03 | 1.13(0.99,1.29) | 1.13(0.98,1.29) | 1.13(0.99,1.30) | 1.13(0.99,1.30) |
|  | Lag04 | **1.19*(1.03,1.38)** | **1.19*(1.02,1.37)** | **1.19*(1.03,1.38)** | **1.19*(1.03,1.38)** |
|  | Lag05 | **1.24*(1.06,1.45)** | **1.23*(1.05,1.44)** | **1.24*(1.06,1.45)** | **1.24*(1.06,1.46)** |
|  | Lag06 | **1.25*(1.06,1.48)** | **1.25*(1.06,1.48)** | **1.26*(1.06,1.49)** | **1.27*(1.07,1.50)** |
| CO | Lag0 | **0.92*(0.84,1.00)** | 0.92(0.85,1.00) | 0.92(0.85,1.01) | 0.92(0.85,1.01) |
|  | Lag1 | 0.99(0.91,1.07) | 0.99(0.92,1.07) | 0.99(0.92,1.08) | 0.99(0.92,1.07) |
|  | Lag2 | 1.07(0.99,1.15) | 1.07(1.00,1.15) | 1.07(1.00,1.16) | 1.07(1.00,1.15) |
|  | Lag3 | 1.04(0.97,1.13) | 1.05(0.97,1.13) | 1.05(0.97,1.13) | 1.05(0.97,1.13) |
|  | Lag4 | 1.05(0.97,1.13) | 1.05(0.97,1.14) | 1.06(0.98,1.14) | 1.06(0.98,1.14) |
|  | Lag5 | 1.02(0.94,1.10) | 1.02(0.94,1.10) | 1.02(0.95,1.11) | 1.03(0.95,1.11) |
|  | Lag6 | 1.00(0.93,1.09) | 1.01(0.93,1.09) | 1.01(0.93,1.10) | 1.02(0.94,1.10) |
|  | Lag01 | 0.94(0.86,1.03) | 0.95(0.86,1.04) | 0.95(0.87,1.04) | 0.95(0.87,1.04) |
|  | Lag02 | 0.99(0.90,1.09) | 1.00(0.90,1.10) | 1.00(0.90,1.10) | 1.00(0.90,1.10) |
|  | Lag03 | 1.01(0.91,1.13) | 1.02(0.92,1.14) | 1.02(0.92,1.14) | 1.02(0.92,1.14) |
|  | Lag04 | 1.04(0.92,1.16) | 1.05(0.93,1.18) | 1.05(0.93,1.18) | 1.05(0.93,1.18) |
|  | Lag05 | 1.04(0.92,1.18) | 1.05(0.93,1.20) | 1.06(0.93,1.20) | 1.06(0.93,1.21) |
|  | Lag06 | 1.04(0.91,1.19) | 1.05(0.92,1.21) | 1.06(0.93,1.22) | 1.07(0.93,1.22) |
| O_3_ | Lag0 | **0.84*(0.74,0.95)** | **0.84*(0.74,0.95)** | **0.84*(0.74,0.95)** | **0.83*(0.73,0.95)** |
|  | Lag1 | **0.81*(0.71,0.91)** | **0.81*(0.71,0.91)** | **0.81*(0.71,0.91)** | **0.80*(0.71,0.91)** |
|  | Lag2 | **0.85*(0.75,0.95)** | **0.85*(0.75,0.96)** | **0.85*(0.75,0.95)** | **0.85*(0.75,0.95)** |
|  | Lag3 | **0.86*(0.77,0.97)** | **0.86*(0.77,0.97)** | **0.86*(0.77,0.97)** | **0.86*(0.77,0.97)** |
|  | Lag4 | **0.81*(0.72,0.91)** | **0.81*(0.72,0.91)** | **0.81*(0.72,0.91)** | **0.81*(0.72,0.91)** |
|  | Lag5 | **0.82*(0.73,0.92)** | **0.82*(0.73,0.92)** | **0.82*(0.73,0.92)** | **0.82*(0.72,0.92)** |
|  | Lag6 | **0.84*(0.75,0.94)** | **0.84*(0.75,0.94)** | **0.84*(0.74,0.94)** | **0.83*(0.74,0.94)** |
|  | Lag01 | **0.79*(0.69,0.91)** | **0.79*(0.69,0.91)** | **0.79*(0.69,0.91)** | **0.79*(0.69,0.90)** |
|  | Lag02 | **0.78*(0.68,0.90)** | **0.78*(0.68,0.90)** | **0.78*(0.68,0.90)** | **0.78*(0.68,0.90)** |
|  | Lag03 | **0.78*(0.67,0.90)** | **0.78*(0.68,0.90)** | **0.78*(0.67,0.90)** | **0.78*(0.67,0.90)** |
|  | Lag04 | **0.76*(0.65,0.88)** | **0.76*(0.66,0.88)** | **0.76*(0.65,0.88)** | **0.76*(0.65,0.88)** |
|  | Lag05 | **0.75*(0.64,0.87)** | **0.75*(0.64,0.87)** | **0.75*(0.64,0.87)** | **0.74*(0.64,0.87)** |
|  | Lag06 | **0.74*(0.64,0.87)** | **0.74*(0.64,0.87)** | **0.74*(0.64,0.87)** | **0.74*(0.63,0.86)** |

Notes: * and bold indicates *p* value <0.05; df, degree of freedom. The interquartile range (IQR) of daily concentrations of PM_2.5_, PM_10_, SO_2_, NO_2_, CO, and O_3_ were 77.50μg/m^3^, 88.94μg/m^3^, 17.40μg/m^3^, 28.36μg/m^3^, 0.88mg/m^3^ and 64.15μg/m^3^, respectively.
